# Clopidogrel in a combined therapy with anticancer drugs—effect on tumor growth, metastasis, and treatment toxicity: Studies in animal models

**DOI:** 10.1371/journal.pone.0188740

**Published:** 2017-12-05

**Authors:** Agnieszka Denslow, Marta Świtalska, Joanna Jarosz, Diana Papiernik, Kseniia Porshneva, Marcin Nowak, Joanna Wietrzyk

**Affiliations:** 1 Department of Experimental Oncology, Hirszfeld Institute of Immunology and Experimental Therapy, Polish Academy of Sciences, Wroclaw, Poland; 2 Faculty of Veterinary Medicine, Wroclaw University of Environmental and Life Sciences, Wroclaw, Poland; Cellcuity, UNITED STATES

## Abstract

Clopidogrel, a thienopyridine derivative with antiplatelet activity, is widely prescribed for patients with cardiovascular diseases. In addition to antiplatelet activity, antiplatelet agents possess anticancer and antimetastatic properties. Contrary to this, results of some studies have suggested that the use of clopidogrel and other thienopyridines accelerates the progression of breast, colorectal, and prostate cancer. Therefore, in this study, we aimed to evaluate the efficacy of clopidogrel and various anticancer agents as a combined treatment using mouse models of breast, colorectal, and prostate cancer. Metastatic dissemination, selected parameters of platelet morphology and biochemistry, as well as angiogenesis were assessed. In addition, body weight, blood morphology, and biochemistry were evaluated to test toxicity of the studied compounds. According to the results, clopidogrel increased antitumor and/or antimetastatic activity of chemotherapeutics such as 5-fluorouracil, cyclophosphamide, and mitoxantrone, whereas it decreased the anticancer activity of doxorubicin, cisplatin, and tamoxifen. The mechanisms of such divergent activities may be based on the modulation of tumor vasculature *via* factors, such as transforming growth factor β1 released from platelets. Moreover, clopidogrel increased the toxicity of docetaxel and protected against mitoxantrone-induced toxicity, which may be due to the modulation of hepatic enzymes and protection of the vasculature, respectively. These results demonstrate that antiplatelet agents can be useful but also dangerous in anticancer treatment and therefore use of thienopyridines in patients undergoing chemotherapy should be carefully evaluated.

## Introduction

In cancer, a number of interactions between the activated vascular endothelial cells, cancer cells, and platelets might cause an imbalance in hemostasis. Platelets can be activated by cancer cells in a process called tumor cell-induced platelet aggregation (TCIP). Studies have shown that tumor cells activate platelets by direct interaction [1] and indirect interaction *via* adenosine diphosphate (ADP), thromboxane A2, or metalloproteinases [2]. In addition, tumor cells can secrete thrombin or stimulate pro-thrombotic activity in other tissues thereby contributing to an increased activity of blood platelets [3].

Maintained platelet activity can promote tumor growth and metastasis in several ways. The platelets secrete mitogenic factors and can stimulate tumor cell proliferation *in vitro* [4] and *in vivo* [5]. In addition, platelets’ granule content may increase survival rate of cancer cells and contribute to the chemoresistance of tumors [6]. Tumor-supporting platelet activity is further enhanced by the pro-angiogenic activity of platelets [7]. Accordingly, inhibition of platelet activity may result in reduced tumor angiogenesis [8] and can therefore inhibit cancer metastasis [9]. Furthermore, inhibition of pro-angiogenic platelet function may lead to an increased permeability of blood vessels within the tumor and by increasing the bioavailability of the drug, enhance the activity of cytotoxic chemotherapy [10].

Recently published studies indicate that platelets directly contribute to an invasive potential of tumor cells. Activated platelets, being an important source of transforming growth factor β (TGF-β), can induce epithelial-to-mesenchymal transition (EMT), which may occur either due to platelet extravasation and their interaction with the tumor cells *in situ* [11] or due to the occurrence of complexes with migrating cells formed in the blood vessel that maintain the invasive potential of tumor cells [12,13]. Interactions of activated platelets with tumor cells promote the adhesion of the latter to the vascular endothelial cells in the target tissue, which precedes the process of extravasation and eventual invasion into the colonized tissues. The molecular mechanism mediating this process is based primarily on selectin-mediated interactions [14,15], however, other factors of platelet origin, such as platelet activating factor (PAF) have also been shown to facilitate extravasation [16,17]. Finally, platelets contribute to pre-metastatic niche formation and thus indirectly favor cancer cells extravasation [12].

ADP is released from the dense bodies of activated platelets, from the damaged red blood cells, and from the vascular endothelial cells. ADP further activates platelets by binding to two receptors: P2Y1 (activating phospholipase C) and P2Y12 (inhibiting the formation of cAMP). Once ADP is bound to its receptors, a change in shape of platelets is initiated from disk to the spherical shape. In addition, an increased activity of phospholipase C followed by the accumulation of cytosolic Ca^2+^ and inhibition of cAMP occurs. In order to initiate such activation, binding of ADP to both of its receptors is required [18]. Because tumor cells activate platelets based on ADP-dependent mechanism [2], an attempt to antagonize the receptors of P2Y1 and P2Y12 or to neutralize ADP molecules appears to be a reasonable antimetastatic strategy. Research on the activity of P2Y1 receptor inhibitors has shown its undeniable role in platelet aggregation, but because of its widespread prevalence in different tissues of the body, its use in the clinical setting is difficult [19]. Therefore, most of the studies are currently focusing on P2Y12 receptor antagonists [19], such as clopidogrel. Clopidogrel, a thienopyridine compound, is a prodrug activated by liver cytochrome enzymes and possesses proven antithrombotic activity. It is widely used in patients at risk of developing thrombosis [20]. Recent studies have also confirmed that inhibition of platelet activity *via* P2Y12-dependent mechanism can reduce the development of metastatic pancreatic cancer [21]. In addition, clopidogrel was found to reduce the risk of metastasis in stage IV colon cancer [22] and in prostate cancer after radiation therapy [23].

Platelet aggregation that accompanies malignant cancer progression is an important factor facilitating both tumor growth and metastasis. Inhibition of platelet activity can presumably contribute to an increased amount of exposure of tumor cells to cytotoxic agents as in blood flow as in tumor tissue. Moreover, inhibition of platelets leads to an incomplete pre-metastatic niche formation, which does not provide factors that are necessary for successful tissue colonization. Therefore, we aimed to analyze the impact of clopidogrel on the anticancer activity of various anticancer agents and to investigate the possible efficacy as well as toxicity of such treatment strategy *in vivo*, in murine, and in human tumor models of breast, prostate, and colon cancer.

## Materials and methods

### Compounds

Clopidogrel was isolated from Plavix (Sanofi Aventis) at the Lodz University of Technology, Poland.

Following anticancer drugs were used in this study: cyclophosphamide (CP)—Baxter Oncology GmbH, (Germany); docetaxel (DTX) and mitoxantrone (MTX)—Ak Scientific (USA); 5-fluorouracil (5-FU)—Medac (UK); cisplatin (CDDP)—Teva Pharmaceuticals Poland Sp. z o.o. (Poland); doxorubicin (DOX)—Sigma-Aldrich Chemie GmbH (Germany); tamoxifen (TMX)—Ebewe Pharma (Austria).

### Mice

In this study, we used 7/8-week-old BALB/c and C57BL/6 female mice purchased from the Center of Experimental Medicine, Medical University of Bialystok, Poland. In addition, 7/8-week-old BALB/c nude female and male mice were provided by Charles Rivers Laboratories (Germany). All animal experiments were performed in the Animal Facility of Institute of Immunology and Experimental Therapy by the staff that meets the required law experience in animal care and research (approval number: 0047). All experiments were performed according to the *Interdisciplinary Principles and Guidelines for the Use of Animals in Research*, *Marketing and Education* issued by the New York Academy of Sciences’ Ad Hoc Committee on Animal Research and European Union rules. Experimental procedures were approved by the first Local Committee for Experiments with the Use of Laboratory Animals, Wroclaw, Poland (permission number: 22/2009, actualized by 32/2011 and 3/2013). Animal care staff performed the monitoring of animals daily: the changes in behavior, posture, and appearance. Mice were humanely euthanized by cervical dislocation if their body weights dropped more than 20% of the original weight for two consecutive measurements, if more than 30% of the original weight remained for one measurement, if their tumor grew larger than 2000 mm^3^ or were necrotic, or if mice were in a state of prostration. Remaining animals were euthanized as indicated in the Table 1 on the day of termination of the experiment. The summary of the number of animals used and euthanized prior to the day of termination of experiments is shown in appropriate figure legends. No animals were found dead in this study. Data on tumor growth and body weights during experiment course for individual mice are included in Supporting Information S1 File or in suitable figures.

**Table 1:** Dosing and treatment schedules used in experiments.

Experimental model	Drug	Single dose	Treatment regimen	DT
**4T1**	Clopidogrel	10 mg/kg	From day 7	
	5-Fluorouracil	35 mg/kg	From day 7*, 5 doses given every second day	26
	Cyclophosphamide	25 mg/kg	From day 7, thrice a week	31
	Doxorubicin	1 mg/kg	From day 8, thrice a week	29
	Cisplatin	0.6 mg/kg	Every day, on days 7–13 and 17–23	26
	Tamoxifen	25 mg/kg	On days 1–2, 5–9 and 15–19	29
**PC-3M-luc2**	Clopidogrel	10 mg/kg	From day 15	
	Docetaxel	10 mg/kg	On days 15 and 22	46
	Mitoxantrone	3 or 1 mg/kg	On days 15, 19, and 23 or 15, 22, and 29	37 or 47
**MC38/EGFP**	Clopidogrel	10 mg/kg	From day 26	
	5-Fluorouracil	35 mg/kg	From day 26, 10 doses given every second day	53
**HT-29-luc2**	Clopidogrel	10 mg/kg	From day 13 day	
	5-Fluorouracil	35 mg/kg	From day 13, every second day	51

*after tumor cell transplantation;

DT—day of experiment termination- euthanasia of all mice; day 0—day of tumor transplantation.

### Cells

Mouse mammary adenocarcinoma 4T1 cells were obtained from the American Type Culture Collection (ATCC, USA). Cells were cultured in RPMI 1640 (IIET, Poland) with Opti-MEM^®^ (Life Technologies, USA) (1:1 v/v) medium with 5% fetal bovine serum (HyClone, Thermo Fisher Scientific Inc., UK), supplemented with 4.5 g/L glucose, 2 mM glutamine, 1.0 mM sodium pyruvate (all from Sigma-Aldrich, Germany).

MC38/EGFP mouse colon cancer cells transduced with green fluorescent protein gene were obtained from the Institute of Immunology and Experimental Therapy, Wroclaw, Poland [24]. MC38/EGFP cell line was cultured *in vitro* in RPMI-1640 medium (IIET, Wroclaw, Poland) supplemented with 1 mg/mL geneticin (Gibco, UK), 2 mM L-glutamine, 1 mM sodium pyruvate (both from Sigma-Aldrich, Germany), 5% fetal bovine serum (HyClone, Thermo Fisher Scientific Inc., UK).

Human prostate cancer cell line (PC-3M-luc2) and colon cancer cell line (HT-29-luc2) stably expressing the firefly luciferase gene were obtained from Caliper Life Sciences Inc. (USA). Cells were cultured in RPMI 1640+Gluta-MAX^™^ medium (Life Technologies, USA) supplemented with 10% fetal bovine serum (Sigma-Aldrich, Germany).

All culture media were supplemented with 100 U/mL penicillin and 100 μg/mL streptomycin (both from Polfa Tarchomin S.A. Warsaw, Poland). Cell cultures were maintained at 37°C in a humidified atmosphere with 5% CO_2_.

Irrespective of the tumor model tested, all cells were trypsinized (IIET, Poland), centrifuged (200 g, 4°C, 5 min), and counted prior to transplantations.

### Experimental design

#### Cell transplantation

For an orthotopic model of mammary gland cancer, 4T1 cells were suspended in Hank’s Balanced Salt Solution (HBSS; IIET, Poland) such that a suspension of 3 × 10^5^ 4T1 cells in 0.05 mL of HBSS was inoculated into the mammary fat pad of female BALB/c mice.

The MC38/EGFP colon cancer cells derived from *in vitro* culture were inoculated subcutaneously (*s*.*c*.) in the right flank region with 1 × 10^6^ cells suspended in 0.2 mL saline per C57BL/6 mouse.

For surgical procedures, mice were intraperitoneally injected with ketamine at a dose of 50 mg/kg (VET-AGRO Sp. z o.o., Poland) and then anesthetized with the mixture of air and isoflurane (3% v/v). For an orthotopic model of prostate tumor, 1.0 cm wide abdominal wall incision was made just above the bladder of male BALB/c nude mice, and the prostate gland was exposed for the injection. Then, 5 × 10^6^ PC-3M-luc2 cells in 0.05 mL of HBSS were inoculated into the dorsal prostate lobes of mice. Immediately after transplantation, incised abdominal wall and skin were sewed with soluble surgical suture (Dexon-“S,” Polfa, Poznań, Poland). For the model of colon cancer metastasis, HT-29-luc2 colon cancer cell suspension (4.5 × 10^6^/50 μL) was inoculated intrasplenically (*i*.*s*.) into female BALB/c nude mice. In brief, anesthetized mouse was placed on a wooden board in the right lateral position, and the abdomen wall was incised at the left subcostal region. The lower pole of the spleen was exposed, and 0.33 × 13 mm needle was inserted at the upper splenic pole. After implantation, the peritoneum and the abdominal wall were sutured with soluble surgical sutures (Dexon-“S,” Polfa, Poznań, Poland).

#### Drug administration

Clopidogrel at a dose of 10 mg/kg/day was administered in sterile drinking water. Prior to treatment initiation, average daily water intake and mouse body weight were determined. The concentration of clopidogrel solution given to mice was calculated according to the formulas

$$\begin{array}{cc} m_{CLO}=\frac{m_{m}\;x\;d}{1000} & C=\frac{m_{CLO}}{V}=\frac{m_{m}\times d}{1000\times V} \end{array}$$

where

*m*_CLO_—amount of clopidogrel [mg] to be administered in a daily cycle to mice

*m*_m_—the average body weight of a mouse [g]

*d*—dose of clopidogrel [mg/1000 g]

*C*—concentration of the solution [mg/mL]

*V*—the average volume of water consumed daily by mouse [mL]

With an exception of DTX, all anticancer drugs were prepared in sterile water for injection. DTX was diluted in a mixture of cremophor (Sigma-Aldrich Hemie GmbH, Steinheim, Germany) and 96% ethanol (0.36:0.64 mL, respectively). The resulting 10× concentrated stock solution was further diluted in saline and immediately injected (0.9% NaCl, IIET, Wroclaw, Poland). All anticancer drugs were administered intraperitoneally in an injection volume of 10 μL/g of body weight and according to the dosages and regimens summarized in Table 1.

### Estimation of the antitumor activity

When subcutaneous (MC38/EGFP) or mammary gland (4T1) tumors became palpable, their maximum length and width were measured thrice a week, and the tumor volume was calculated according to the formula:

$$TV=\frac{1}{2}\times a^{2}\times b$$

where *TV* is the tumor volume, *a* is the shorter diameter, and *b* is the longer diameter.

*In vivo* visualizations of PC-3M-luc2 or HT-29-luc2 tumors growing in prostate gland or spleen of BALB/c nude mice were performed using an In-vivo MS FX PRO system (Carestream Health INC., USA), no more often than every 4 days starting from the 15th or 13th day of the experiment, respectively. In brief, about 10 min before imaging, D-luciferin potassium salt (Synchem INC., Germany) was intraperitoneally administered to each mouse at a dose of 150 mg/kg. Then, animals were anesthetized with a 3–5% (v/v) mixture of isoflurane (Forane, Abbott Laboratories, USA) in synthetic air (200 mL/min). Anesthesia was maintained with 1.5–2% (v/v) mixture of isoflurane and synthetic air delivered via individual masks. Visualization was performed using the following settings: for X-ray—t = 2 min, f-stop = 5.57, FOV = 198.6; for luminescence capture—t = 3 min, binning 2×2, f-stop = 5.57, FOV = 198.6. Images were analyzed with Carestream MI SE software (Carestream Health INC., USA). The intensity of the luminescent signal is presented as the sum intensity of the region of interest and expressed in arbitrary units (a.u.).

Tumor tissue was also excised and weighed on the last day of the experiment (Table 1).

### Evaluation of the antimetastatic activity

To determine the antimetastatic activity of the compounds in 4T1 mammary gland model, the lungs of tumor-bearing mice were excised, weighed, and transferred into 5% solution of buffered formalin. After tissue fixation, metastatic foci were visually counted.

To detect metastases in the mice with prostate cancer, their livers, lungs, kidneys, bones and axillary, as well as inguinal lymph nodes were isolated and fixed in buffered formalin on the day of the necropsy. All isolated tissues were paraffin embedded and cut into 4 μm thick sections that were later dewaxed with xylene and rehydrated in a gradient of ethanol. Finally, sections were washed in distilled water and cytoplasm was stained with eosin while nuclei were counterstained in hematoxylin. Such preparations were next dehydrated in an alcohol gradient and coverslip mounted. The number of metastases in isolated tissues was counted at 50× or 400× magnitude.

### Evaluation of tumor angiogenesis

Tumor angiogenesis was analyzed by imaging of tumor blood vessel permeability or immunohistochemistry staining of platelet endothelial cell adhesion molecule (PECAM-1; CD31). For imaging, an IRDye 800CW PEG Contrast Agent (LI-COR, Lincoln, USA) was prepared according to the manufacturer’s instructions. The contrast agent was injected intravenously (*i*.*v*.) at a dose of 1 nmol/100 μL/mouse. After 24 h, animals were anesthetized with the 3–5% (v/v) mixture of isoflurane (Forane, Abbott Laboratories, USA) in synthetic air (200 mL/min) and placed on an animal handling station equipped with an individual mask providing with a 1.5–2% (v/v) mixture of isoflurane and synthetic air (In Vivo Ms FX Pro, Carestream Health INC., USA). Visualization was performed using the following settings: for X-Ray t = 2 min, f-stop = 5.57, FOV = 198.6; for fluorescence capture t = 30 s, f-stop = 2.8, FOV = 141 (induction: 760 nm, emission: 830 nm). Images were analyzed with Carestream MI SE software (Carestream Health INC., USA). The recorded fluorescence signal is presented as the sum intensity for each region of interest and expressed in a.u.

Immunohistochemistry was performed in tumors isolated from mice on the last day of experiment. In brief, tissues were fixed in buffered formalin and then cut into 4 μm thick sections that were later dewaxed with xylene and rehydrated in a gradient of ethanol. For antigen retrieval, sections were heated in a water bath at 96°C for 20 min with EnVision^™^ FLEX Target Retrieval Solution, High pH (Dako^®^, Carpinteria, USA). Endogenous peroxidase activity was quenched in EnVision^™^ FLEX Peroxidase-Blocking Reagent for 5 min. Thereafter, sections were incubated with primary antibody against PECAM-1 (diluted in 1:50 ratio) and washed in EnVision^™^ FLEX Wash Buffer. Then, EnVision^™^ FLEX/HR SM802 detection reagent was added for 30 min at room temperature. The reaction was developed using 3,3-diaminobenzidine tetrahydrochloride solution (DAB, EnVision^™^ FLEX DAB+Chromogen (DAKO^®^, Carpinteria, USA). Finally, sections were washed in distilled water and nuclei were counterstained in hematoxylin. Then, preparations were dehydrated in an alcohol gradient and coverslip mounted. Mean vessel density was evaluated by counting PECAM-1-positive vessels in five different fields of view at 200× magnitude.

### Platelet activation status

Blood samples were collected during necropsy into tubes containing 0.05 mL of 5% ethylenediaminetetraacetic acid (EDTA) solution (Sigma-Aldrich, Germany). Platelet morphology was analyzed using Mythic 18 analyzer (C2 Diagnostics, France). Blood plasma was obtained by centrifugation (2000 × *g*, 15 min, 4°C) and stored at −80°C until further analysis. Prostacyclin generation was determined by the quantification of plasma 6-keto-prostaglandin F1α (6-keto-PGF1α) levels. Based on thromboxane B_2_ (TXB2), von Willebrand factor (vWF), and soluble P-selectin plasma concentrations, platelet activation status was estimated. Using commercial kits available from Cusabio Biotech Co. Ltd. (Wuhan, China), all analyzes were conducted by using ELISA technique. In addition, plasma concentration of TGF-β1 was determined with ELISA kit from Boster Biological Technology (USA). All ELISA-based analyzes were conducted according to the manufacturer’s instructions.

### Protein expression in tumor tissue

Protein expression in prostate tumor tissue was analyzed according to the standard western blot procedure [25]. In brief, tumor tissue samples were collected and immediately frozen on the last day of the experiment. The samples were homogenized in RIPA buffer (Sigma-Aldrich, Germany) using a FastPrep^®^-24 MP Bio device (Mp Biomedicals LLC., USA) with the following settings: CP 24 × 2, 6 m/s, 40 s. Protein content in all samples was analyzed using a Bio-Rad Protein Assay (Bio-Rad Laboratories Inc., USA) according to the manufacturer’s protocol. Samples containing 100 μg of protein were separated on the pre-cast 4–20% gradient gels (Bio-Rad Laboratories, Inc., USA) and transferred onto 0.45 μm polyvinylidene fluoride (PVDF) membranes (Merck Millipore, USA). Next, the membranes were probed with primary rabbit polyclonal anti-E-cadherin (1:1000) and anti-N-cadherin (1:1000) antibodies (all from Proteintech Group, USA) or mouse anti-β-actin (1:1000, Sigma-Aldrich, Germany) antibody. Finally, the analyzed proteins were detected with IRDye^®^ 800CW Goat anti-Rabbit IgG or IRDye^®^ 680RD Donkey anti-Mouse IgG (both from LI-COR, USA) according to the manufacturer’s instructions. Blots were visualized in ODDYSEY^®^ CLx Imager (LI-COR, USA) and analyzed with ImageJ Software as follows. The total E-cadherin cellular content comprising truncated and unprocessed E-cadherin (with a molecular weight of approximately 100 and 130 kDa, respectively) was calculated. Similarly, total cellular content of N-cadherin comprising mature and unprocessed N-cadherin (with a molecular weight of approximately 70 and 100 kDa, respectively) was determined. Then, E-cadherin and N-cadherin contents were normalized to β-actin. Finally, E-cadherin to N-cadherin ratios in individual samples were calculated and presented as mean ± standard error of mean (SEM) values.

### Toxicity of the anticancer treatment

The toxicity of the proposed anticancer treatment approach and its effect on the overall health condition were estimated based on body weight changes as well as morphological and biochemical analysis. The body weight of experimental animals was measured thrice each week throughout the course of our experiments. The changes in body weight are presented in supplementary materials (S1, S2, S4, S7, S9, S11 and S13 Figs). The health of the mouse was monitored daily. In case of the observed toxicity, animals demonstrating an obvious deterioration in health status were euthanized immediately, and the survival curves were plotted.

Blood morphology was performed with Mythic 18 analyzer (C2 Diagnostics, France). Biochemical analyzes were performed in Cobas C 111 analyzer (Roche Diagnostics, Switzerland) using reagents and procedures provided by the manufacturer.

### Statistical analysis

Data normality was estimated using the Shapiro–Wilk test with a predetermined value of *p*<0.05. The Tukey–Kramer multiple comparisons test for parametric data or the Kruskal–Wallis Test for multiple comparisons for nonparametric data was applied; *p* values lower than 0.05 were considered significant. All calculations were performed using GraphPad Prism 7 (GraphPad Software, Inc., USA) software.

## Results

### The effects of clopidogrel in the combination anticancer therapy in the model of 4T1 mouse mammary gland cancer

#### Improvement of 5-FU and CP antitumor and antimetastatic activity by clopidogrel

**Fig 1:**
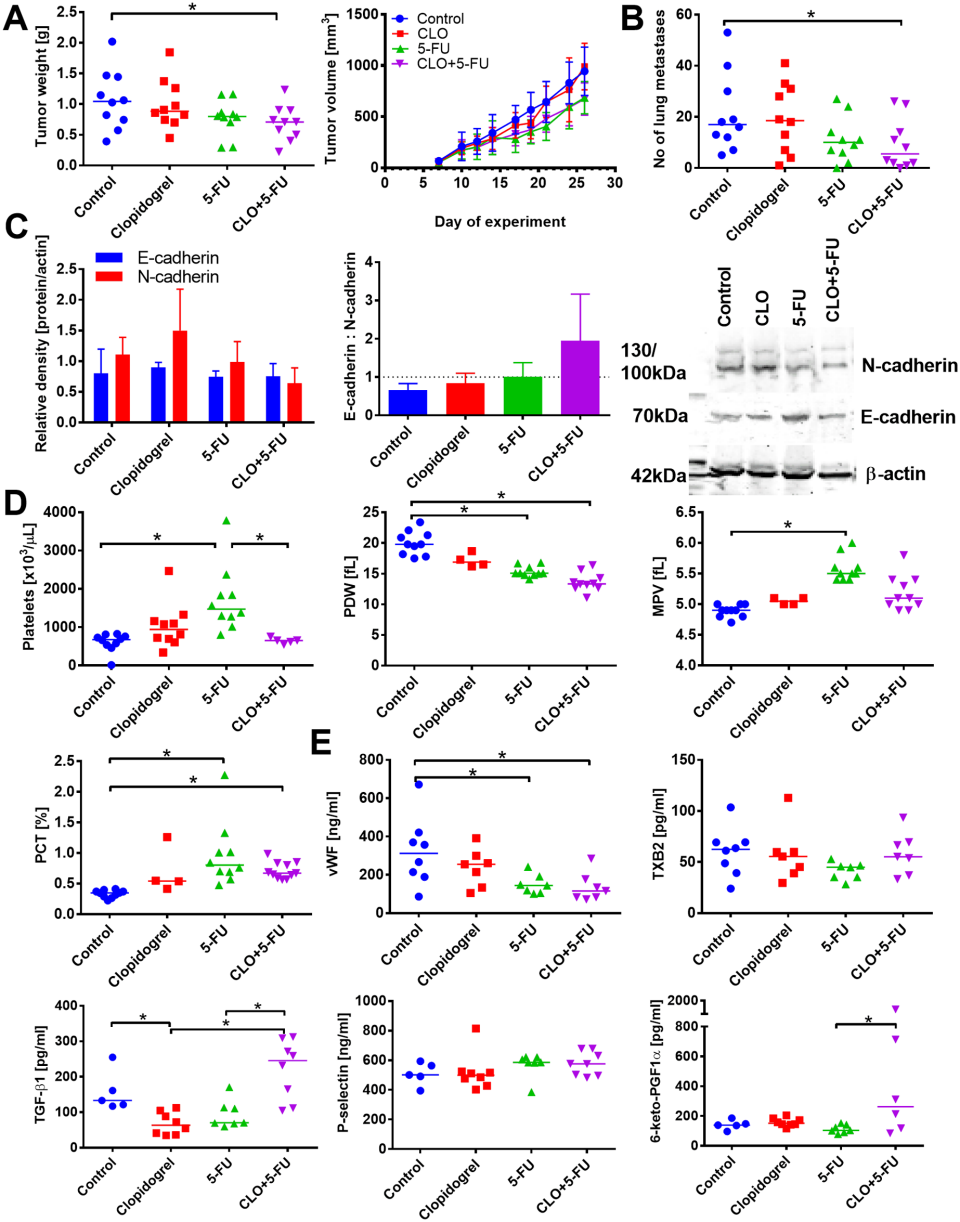
Combination therapy with clopidogrel and 5-fluorouracil (5-FU) reduce the growth and metastasis of 4T1 tumors. (A) Tumor weight on day 26 and kinetics of tumor growth. (B) Number of lung metastatic foci. (C) Expression of E- and N-cadherin in tumor tissue (left graph), E:N-cadherin ratio (middle) and representative blots (right). (D) Platelets’ morphological parameters, including platelet count, platelet distribution width (PDW), mean platelet volume (MPV), and plateletcrit (PCT). (E) ELISA measurements of plasma proteins corresponding to platelets activity: von Willebrant factor (vWF), thromboxane b2 (TXB2), transforming growth factor beta 1 (TGF-β1), P-selectin, and prostacyclin metabolite (6-keto-PGF1α). All graphs show values for individual animals with median line; the exception is panel D: mean with standard error of mean (SEM) and kinetics of tumor growth: mean with standard deviation (SD) is presented. N = 10 mice per group; some tests were performed on tissue or plasma from selected animals from each group (at least 3 in western blot tests; data for individual blots presented in S1 Table). All mice were euthanized on day 26. Statistical analysis: Kruskal–Wallis test for multiple comparisons; *p<0.05. The values of selected morphological parameters of platelets in healthy BALB/c mice: platelet count: 245 ± 95 [×10^3^/μL]; MPV: 6.6 ± 0.3 [fL]; PDW: 47 ± 1 [fL]; PCT: 0.2 ± 0.06 [%]. The level of TGF-β1: 320 ± 449; P-selectin: 100 ± 10 in healthy BALB/c mice.

**Fig 2:**
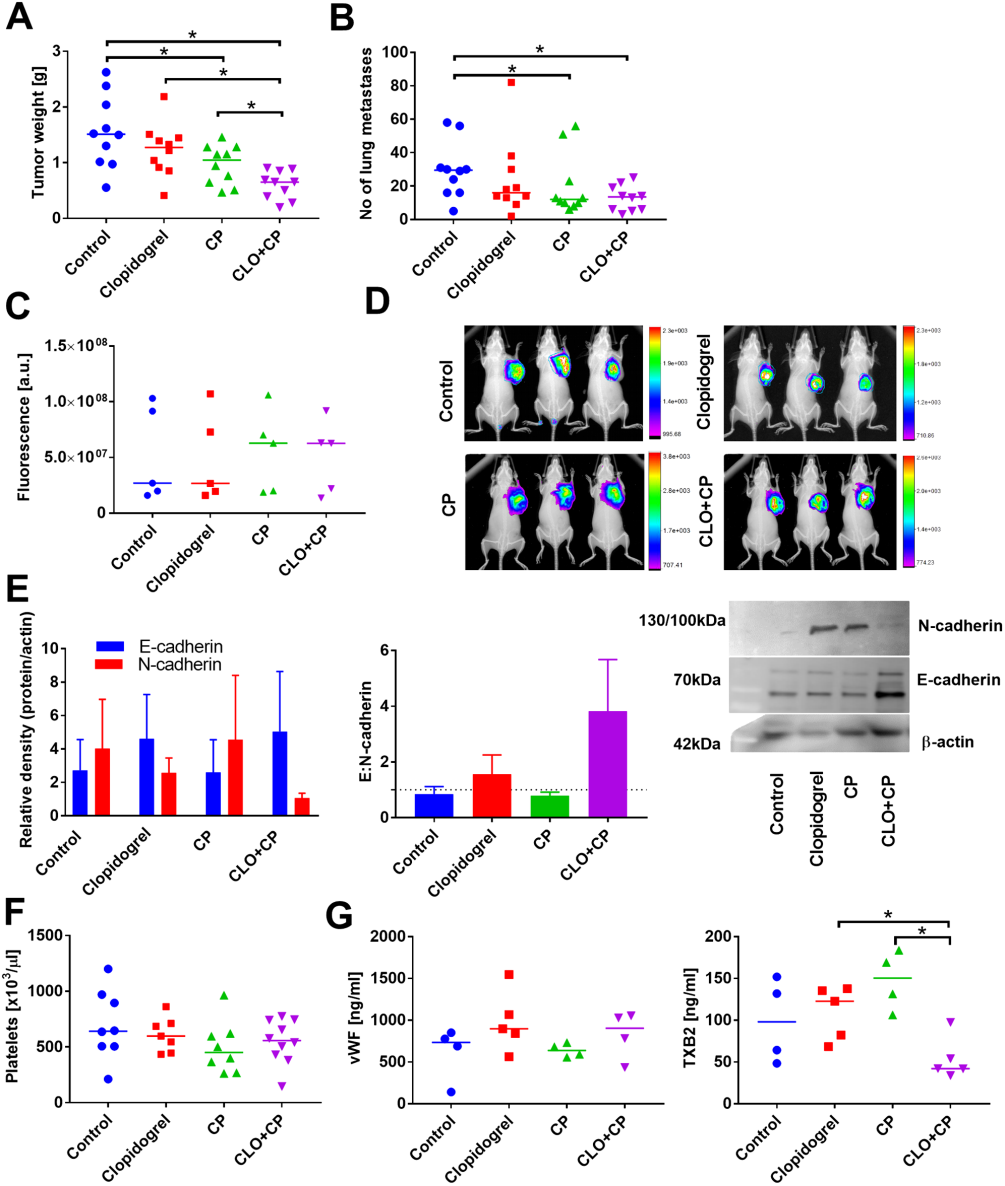
Combination therapy with clopidogrel and cyclophosphamide (CP) reduce the growth and metastasis of orthotopic 4T1 tumors. (A) Tumor weight on day 31 and kinetics of tumor growth. (B) Number of lung metastatic foci. (C) Tumor blood vessel permeability measured as the accumulation of IRDye 800CW PEG Contrast Agent 24 h after injection. (D) Example photographs of fluorescence measurements are presented. (E) Expression of E- and N-cadherin in tumor tissue (left graph), E:N-cadherin ratio (middle), and representative blots (right). (F) Platelet count. (G) ELISA measurements of plasma proteins connected with platelets activity: von Willebrant factor (vWF) and thromboxane B2 (TXB2). All graphs show values for individual animals with median line; the exception is panel (E): mean with standard error of mean (SEM) and kinetics of tumor growth: mean with standard deviation (SD) is presented. N = 10 mice per group; some tests were performed on tissue or plasma from selected animals from each group (at least 3 in western blot tests; data for individual blots presented in S1 Table). All mice were euthanized on day 31. Statistical analysis: Kruskal–Wallis test for multiple comparisons; *p<0.05.

Clopidogrel itself did not affect either the growth of primary 4T1 tumors or their metastatic potential. But, when the drug was used in combination therapy with 5-FU or CP, significant improvement of the antitumor activity was observed (Figs 1A and 2A). In addition, when clopidogrel was included in the treatment schedule, we observed a limited metastatic spread of 4T1 cells as compared to 5-FU (Fig 1B) or CP (Fig 2B) alone.

To determine the possible underlying mechanisms of the enhanced anticancer activity of the combined therapy, we performed a number of subsequent analyses. First, vascular permeability of 4T1 primary tumors was estimated based on the intratumoral accumulation of IRDye 800CW PEG Contrast Agent. As shown in Fig 2C and 2D, clopidogrel alone did not affect tumor blood vessel permeability, whereas CP dosed in metronomic schedule tended to increase it.

Then, we measured the expression of E and N-cadherins in tumor tissue. Neither 5-FU, CP, nor clopidogrel, when used alone, affected the expression of any of these proteins (Fig 1C); however, in mice treated with both clopidogrel and CP or, to a lesser extent with 5-FU, an increase in E:N-cadherin ratio was noticed (Fig 2E).

Clopidogrel and CP when used alone, as well as in the combination therapy, did not affect the platelet count or any other morphological parameters of platelets (Figs 1D and 2F; data shown only for platelet count in the combined treatment with CP). While 5-FU significantly increased platelet count, mean platelet volume (MPV), and plateletcrit (PCT), it decreased platelet distribution width (PDW; Fig 1D). When clopidogrel was combined with 5-FU, these parameters were changed noticeably, leading to a significant decrease in platelet count as compared to 5-FU, accompanied by the reduced values of other platelet morphological parameters (*p*<0.05 as compared to control group but not to 5-FU) (Fig 1D).

Analysis of the selected biochemical parameters related to platelet activity demonstrated that upon single-drug therapy with clopidogrel, plasma level of TGF-β1 significantly decreased only in case of 4T1 tumor-bearing mice. But, plasma levels of TGF-β1 were significantly increased in mice characterized by the lowest number of metastatic foci in animals treated with clopidogrel combined with 5-FU. TGF-β1 levels were increased significantly as compared to clopidogrel and 5-FU administered alone (Fig 1E). When used as a single-drug therapy as well as when combined with clopidogrel, 5-FU (but not CP) decreased plasma level of von Willebrant factor (vWF) (Figs 1E and 2G). Thromboxane B2 (TXB2) level in mice treated with clopidogrel alone or when combined with 5-FU remained unchanged (Fig 1F), but in mice treated with CP alone, the level of TXB2 tended to increase. In mice treated with clopidogrel and CP, plasma concentration of TXB2 was found to be the lowest (*p*<0.05 vs. CP and clopidogrel alone) (Fig 2G). Finally, P-selectin concentration in plasma of mice treated with either clopidogrel or 5-FU given alone or in the combination remained at a level similar to those observed for control tumor-bearing mice (Fig 1F).

Body weight of animals transitorily decreased in animals treated with 5-FU or CP administered alone as well as in the combination with clopidogrel. Clopidogrel alone did not affect the body weight of mice (S1A and S2A Figs).

We also observed that in mice treated with clopidogrel combined with 5-FU, the total leukocyte count significantly increased (S1B Fig) primarily due to an increase in the number of lymphocytes and monocytes. The leukocyte count was also found to be significantly increased in animals treated with clopidogrel as a single-drug therapy or when combined with 5-FU (S1D and S2D Figs). However, in mice treated with clopidogrel in combination with CP, we found a significant decrease in the number of lymphocytes (S2D Fig) that correlated with decreased spleen weight (S3C Fig).

In case of animals treated with clopidogrel combined with both 5-FU and CP, we found a decrease in erythrocyte cell count (*p*<0.05, S1C and S2C Figs). In addition, other morphological parameters of erythrocytes such as mean corpuscular hemoglobin (MCH) and mean corpuscular hemoglobin concentration (MCHC) were found to be significantly decreased in animals treated with clopidogrel, 5-FU, and CP alone or when used as a combination therapy. Moreover, 5-FU used alone or in combination with clopidogrel decreased the level of hemoglobin and increased mean corpuscular volume of erythrocytes (MCV) significantly (S1E Fig). When combined with clopidogrel, CP significantly decreased the level of hemoglobin and MCHC, whereas when used alone or in the combination therapy, it increased the red cell distribution width (RDW) (*p*<0.05, S2E Fig).

**Fig 3:**
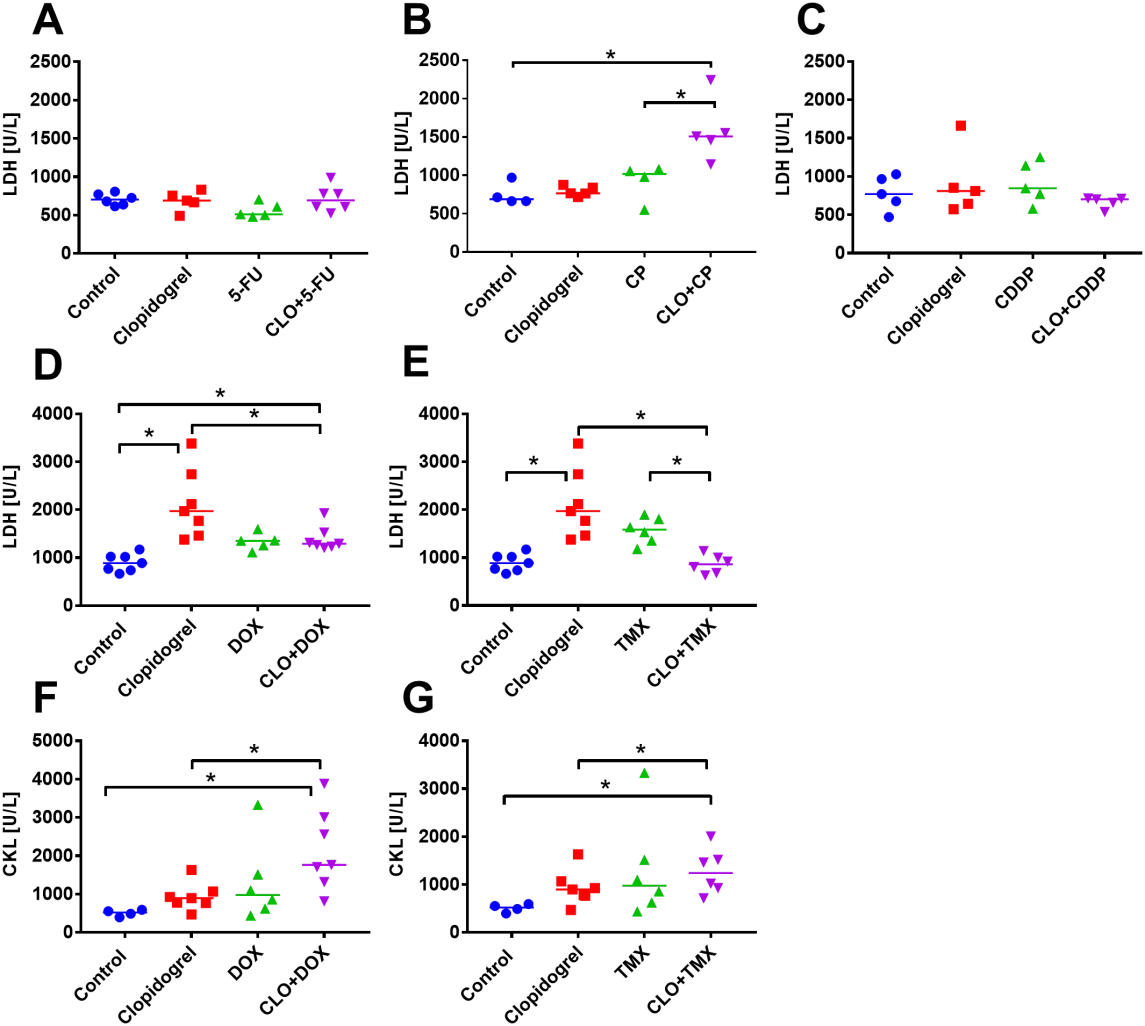
Lactate dehydrogenase (LDH) and creatine kinase (CK) levels in animals treated with clopidogrel combined with 5-fluorouracil (5-FU), cyclophosphamide (CP), cisplatin (CDDP), doxorubicin (DOX), and tamoxifen (TMX) in 4T1 tumor model. LDH values in mice treated with clopidogrel combined with: (A) 5-FU, (B) CP, (C) CDDP, (D) DOX, and (E) TMX. CK values for clopidogrel in the combination therapy with (F) DOX and (G) TMX. All graphs show values for individual animals with median line. Plasma from 4 to 7 mice per group was analyzed. Statistical analysis: Kruskal–Wallis test for multiple comparisons; *p<0.05.

To further analyze the potential toxic effects of the proposed therapeutic regimen, we measured blood biochemical parameters. For the combined treatment, we did not observe any general signs of toxicity such as loss of body weight. However, we noticed an increase in plasma lactate dehydrogenase (LDH) level as compared to control or CP-treated mice (Fig 3B). Such effect was not observed in the combined treatment with 5-FU (Fig 3A). Other parameters, including ASP, ALT, albumin, creatinine, urea, and the weight of selected organs collected during necropsy did not change significantly (S3 Fig).

#### Deterioration of CDDP, DOX, and TMX anticancer activity by clopidogrel

**Fig 4:**
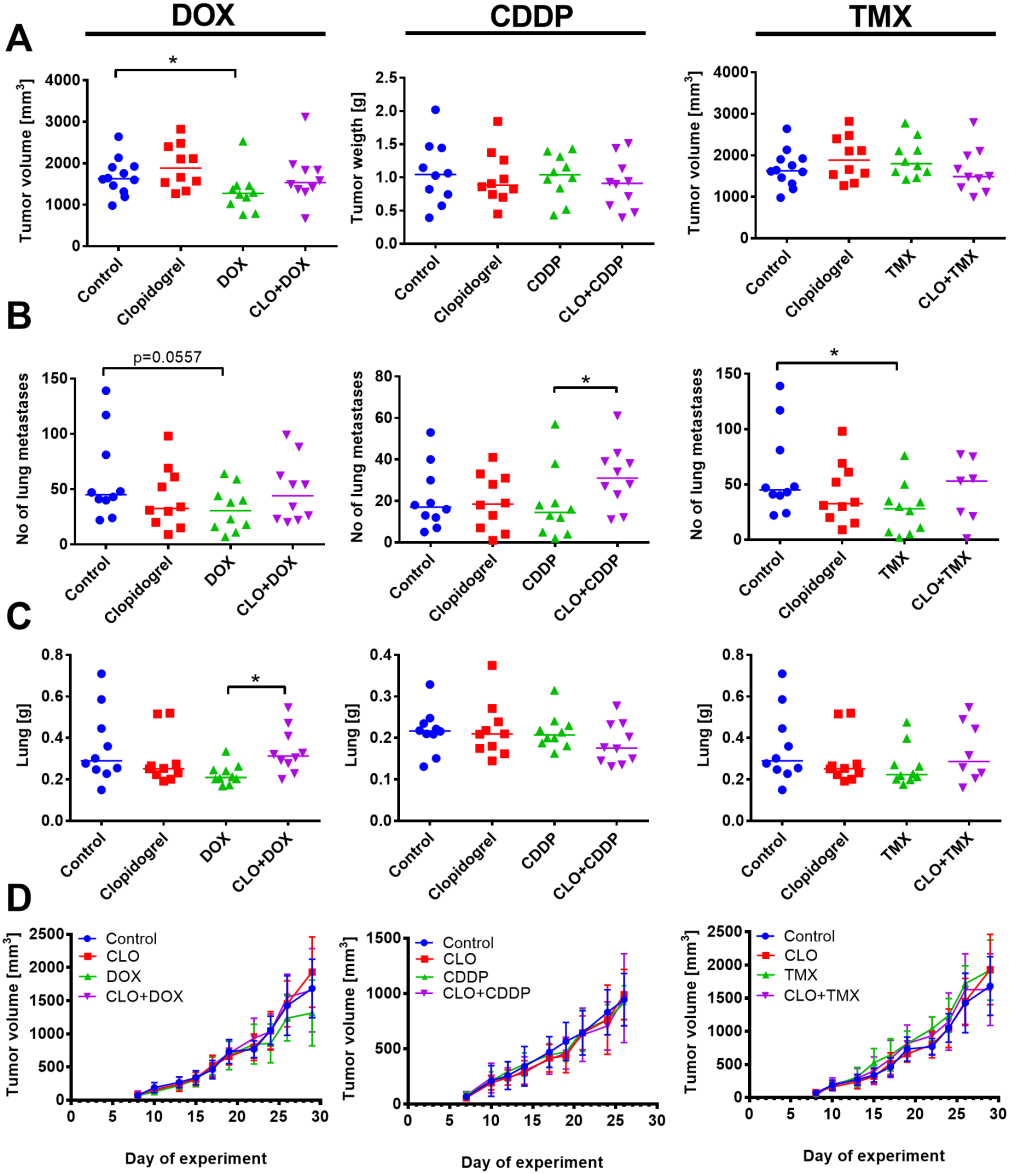
Unfavorable effect of clopidogrel on cisplatin (CDDP), doxorubicin (DOX), and tamoxifen (TMX) anticancer activity in 4T1 mammary gland cancer model. (A) Tumor size on the last day of experiment. (B) Number of lung metastatic foci. (C) Lung weight and (D) kinetics of tumor growth in mice treated with clopidogrel combined with DOX, CDDP, and TMX. All graphs show values for individual animals with median line. The exception is (D): mean with standard deviation (SD) is presented. N = 10–12 mice per group. All mice were euthanized: DOX and TMX experiment on 29, CDDP experiment on day 26. Statistical analysis: Kruskal–Wallis test for multiple comparisons, *p<0.05.

When 4T1 tumor-bearing mice were treated with clopidogrel in combination with DOX, CDDP, or TMX, there was an overall disadvantageous effect. For example, simultaneous administration of DOX and clopidogrel resulted in the loss of significant antitumor and antimetastatic activity of the cytostatic drug. Therefore, we observed an insignificant tumor growth inhibition (Fig 4A) and an increased number of lung metastases (Fig 4B) (*p*<0.05) when compared to DOX alone. In case of both CDDP and TMX, their effect on primary tumor growth was not affected, but their antimetastatic activity was diminished when the drugs were administered with clopidogrel (Fig 4B and 4C).

**Fig 5:**
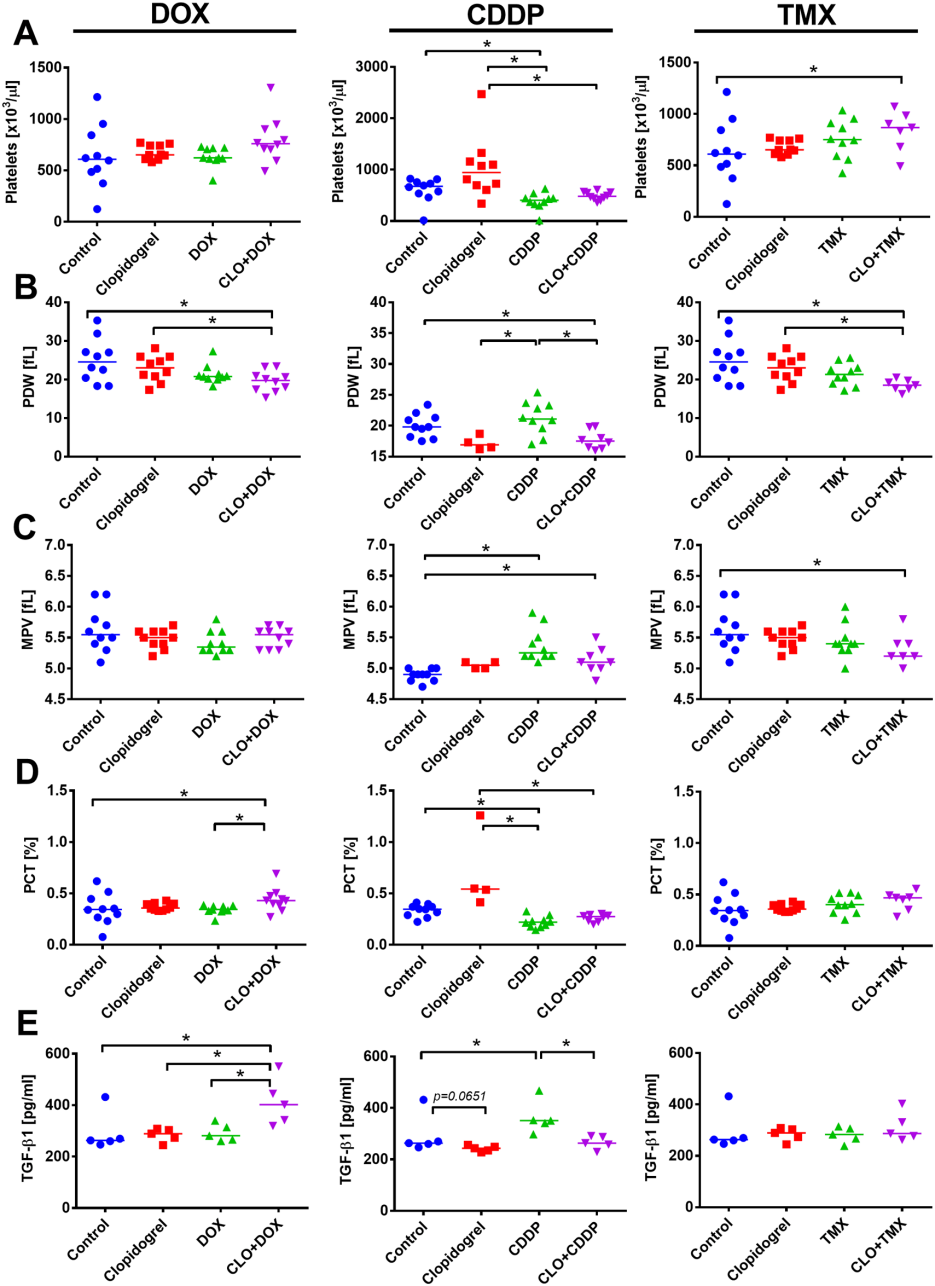
Platelet parameters in mice bearing 4T1 tumors treated with clopidogrel combined with doxorubicin (DOX), cisplatin (CDDP), and tamoxifen (TMX). Morphological parameters of platelets: (A) Platelet count. (B) Platelet distribution width (PDW). (C) Mean platelet volume (MPV). (D) Plateletcrit (PCT). (E) Concentration of transforming growth factor β1 (TGF-β1) in mice plasma. All graphs show values for individual animals with median line. N = 4–10 mice per group. Statistical analysis: Kruskal–Wallis test for multiple comparisons; *p<0.05.

Treatment with DOX (either alone or in combination therapy) did not affect platelet count, but in combination therapy, DOX and clopidogrel decreased PCT value that led to a significant increase in PDW (Fig 5A). In mice treated with CDDP alone or when combined with clopidogrel, platelet count, MPV, and PCT were found to be significantly decreased. PDW that was increased by CDDP treatment was found to be significantly decreased in mice treated with both agents as compared to CDDP alone (Fig 5B). Clopidogrel combined with TMX increased platelet count. Similar to the combined treatment with CDDP or DOX, PDW was found to be significantly decreased in the combined treatment with clopidogrel and TMX. MPV (as in the combined treatment with CDDP) was found to be significantly decreased when mice were treated with combined clopidogrel and TMX (Fig 5C). Plasma TGF-β1 levels from clopidogrel-treated mice was found to be unchanged or decreased. Among the anticancer drugs tested, only CDDP significantly increased the plasma concentration of TGF-β1. In the combined treatment with DOX, clopidogrel significantly increased the level of TGF-β1; however, when combined with CDDP, it significantly decreased the level of TGF-β1 when compared to CDDP alone (Fig 5E).

Combination therapy did not affect body weight of mice significantly as compared to the treatment with DOX, CDDP, or TMX alone (S4A Fig).

In mice treated with clopidogrel and DOX, we observed a significantly higher number of lymphocytes, monocytes, and granulocytes (*p*<0.05 vs. control and DOX-treated animals). This effect was also observed in mice treated with TMX. On the contrary, in mice treated with combination therapy with CDDP, we found the lowest total number of leukocytes resulting from the decreased number of all three leukocyte populations (S4B–S4E Fig).

DOX alone decreased erythrocyte count significantly, and this effect was found to be reversed in the combination therapy (S5A Fig). Similarly, the levels of hemoglobin, hematocrit, MCV, and MCH were found to be elevated in DOX-treated animals, whereas these parameters were found to be decreased when combined with clopidogrel. In addition, clopidogrel administered in combination therapy reversed the increase in MCHC and RDV values observed in mice treated with DOX alone (S5B–S5G Fig). We found analogous but not prominent effects on erythrocyte characteristics during the combined treatment with TMX (S4A–S4G Fig). On the contrary, clopidogrel did not change the effects of CDDP on erythrocyte characteristics (S5A–S5G Fig).

Clopidogrel in combination with DOX or TMX did not affect the weight of the selected internal organs significantly (S6A–S6C Fig). Single-drug therapy with CDDP resulted in significant decrease in the weights of liver and kidneys. Simultaneous administration of clopidogrel with CDDP further decreased weight of the liver (S6A and S6C Fig). LDH activity was either unchanged or increased in the plasma of mice treated with clopidogrel alone (Fig 3C–3E). CDDP did not induce any changes in LDH activity in plasma of the treated animals (Fig 3C). Only in the combined treatment with clopidogrel and TMX, significant decrease in LDH level, as compared to each agent alone, was observed (Fig 3E). In addition, in mice treated with DOX and TMX, we analyzed the level of creatine kinase (CK). Clopidogrel alone tended to increase the level of CK, and in combined treatment with DOX and with TMX, a significant increase in the levels of CK was observed (Fig 3F and 3G).

### Clopidogrel in combination with MTX and DTX in orthotopic murine model of human prostate cancer

#### Protection against MTX-induced toxicity

**Fig 6:**
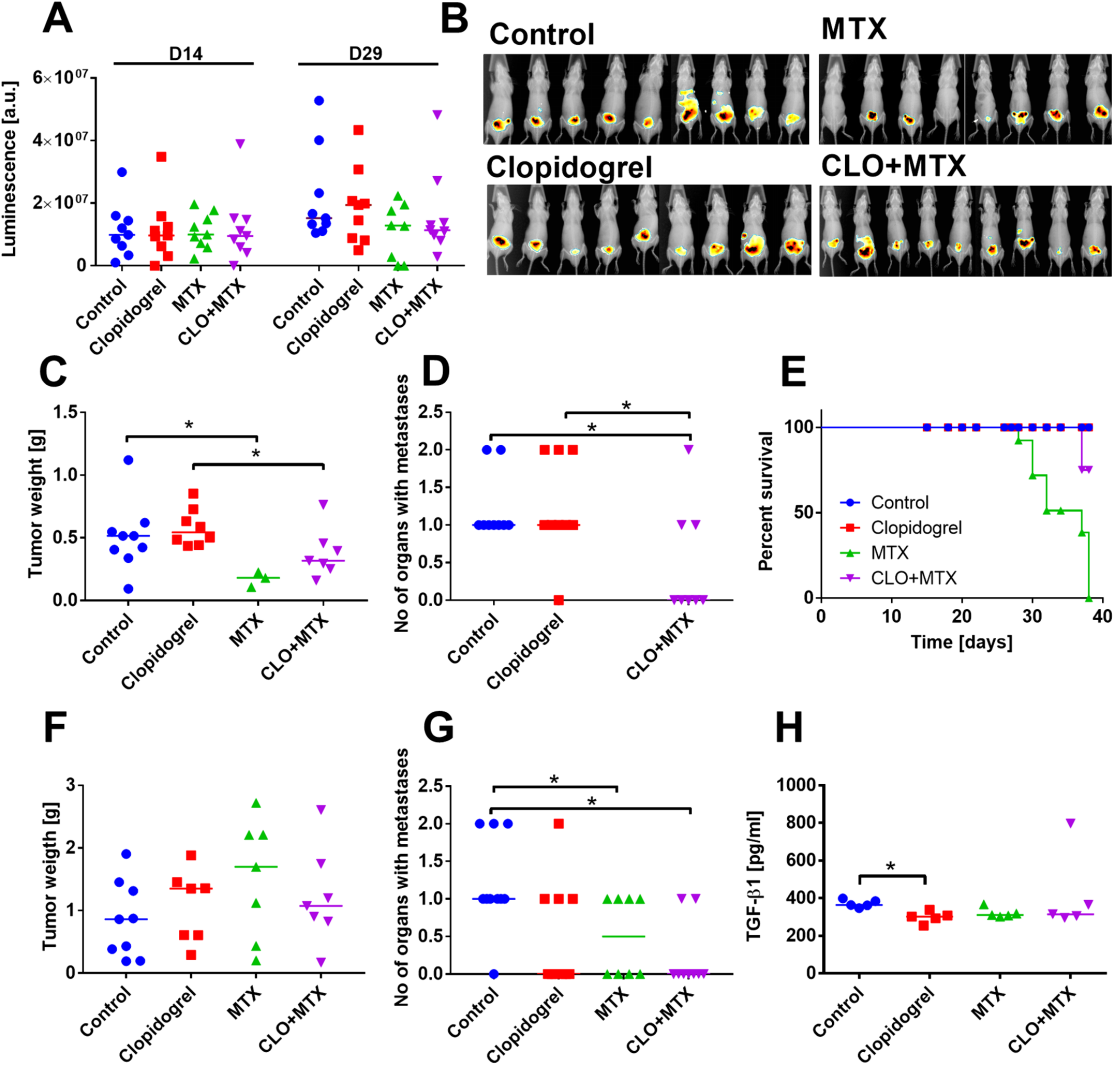
Clopidogrel protects against the toxicity of MTX in mice bearing human PC-3M-luc2 prostate tumors. (A) Luminescence of prostatic tumors on day 14 (a day prior to the treatment initiation) and 29. (B) Photographs of mice on day 29 of experiment. (C) Tumor weight of tumors harvested on day 37. (D) Number of organs with diagnosed metastases. (E) Survival curves. (A)–(E) Data from mice treated with 3 mg/kg/dose of MTX. All mice were euthanized on day 37; the number of animals euthanized before: 10. N = 9 mice per group. (F) Tumor weight of tumors harvested on day 47. (G) Number of organs with diagnosed metastases. (H) Transforming growth factor β1 (TGF-β1) plasma level. (F)–(H) Data from mice treated with 1 mg/kg/dose of MTX. All mice were euthanized on day 47. N = 7–9 mice per group. All graphs show values for individual animals with median line; the exception: (E) shows percent of survival on indicated days. Statistical analysis: Kruskal–Wallis test for multiple comparisons, *p<0.05. Survival analysis: Mantel–Cox test showed significant differences between survival curves (*p*<0.0001).

The growth of orthotopically transplanted PC-3M-luc2 tumors was significantly inhibited in mice treated with MTX (Fig 6A–6C). This high chemotherapeutic effect was accompanied by an increased mortality of the treated animals (Fig 6E). In the combined therapy with clopidogrel, not only the antitumor activity decreased but also the toxic effect of MTX decreased. Platelets’ morphological parameters did not change because of therapy. The tumor progression of mice inoculated with PC-3M-luc2 was accompanied by the loss in body weight that was further enhanced by the treatment with MTX (S7A Fig). Mice treated with both clopidogrel and MTX had significantly lower total leukocyte and lymphocyte count (S7B and S7D Fig), whereas erythrocyte count, hemoglobin, hematocrit, and MCV were not found to be affected by the treatment. Clopidogrel increased MCH and MCHC significantly. RDW was found to be increased in mice treated with combination therapy (S7C and S7E Fig).

In mice treated with clopidogrel and MTX, we found a significant decrease in the weight of liver, kidneys, spleen, and lungs (S7F Fig).

We also performed the experiment with low dose of MTX (1 mg/kg/dose thrice every week). In this experiment, while we did not observe any symptoms of treatment-related toxicity, we also did not observe any antitumor activity of the drug. But, MTX either alone or in combination with clopidogrel, decreased the number of organs with diagnosed metastases significantly (Fig 6F and 6G).

We also analyzed the effect of treatment on tumor angiogenesis; however, we did not observe any significant changes in microvessel density (MVD) (S7G Fig). Plasma level of TGF-β1 was found to be decreased by clopidogrel treatment, but MTX alone and in combination with clopidogrel did not change the level of TGF-β1 significantly (Fig 6H). In this study, MTX was found to increase only urea level significantly (among tested LDH, CK, ALT, AST, creatinine, and urea; S8 Fig); clopidogrel decreased urea level as compared to MTX (S8F Fig). Moreover, in combination therapy with MTX, we found a significant increase in the level of CK (S8B Fig) and creatinine (S8E Fig) (*p*<0.05 vs. clopidogrel).

#### Clopidogrel increases the toxicity of DTX

**Fig 7:**
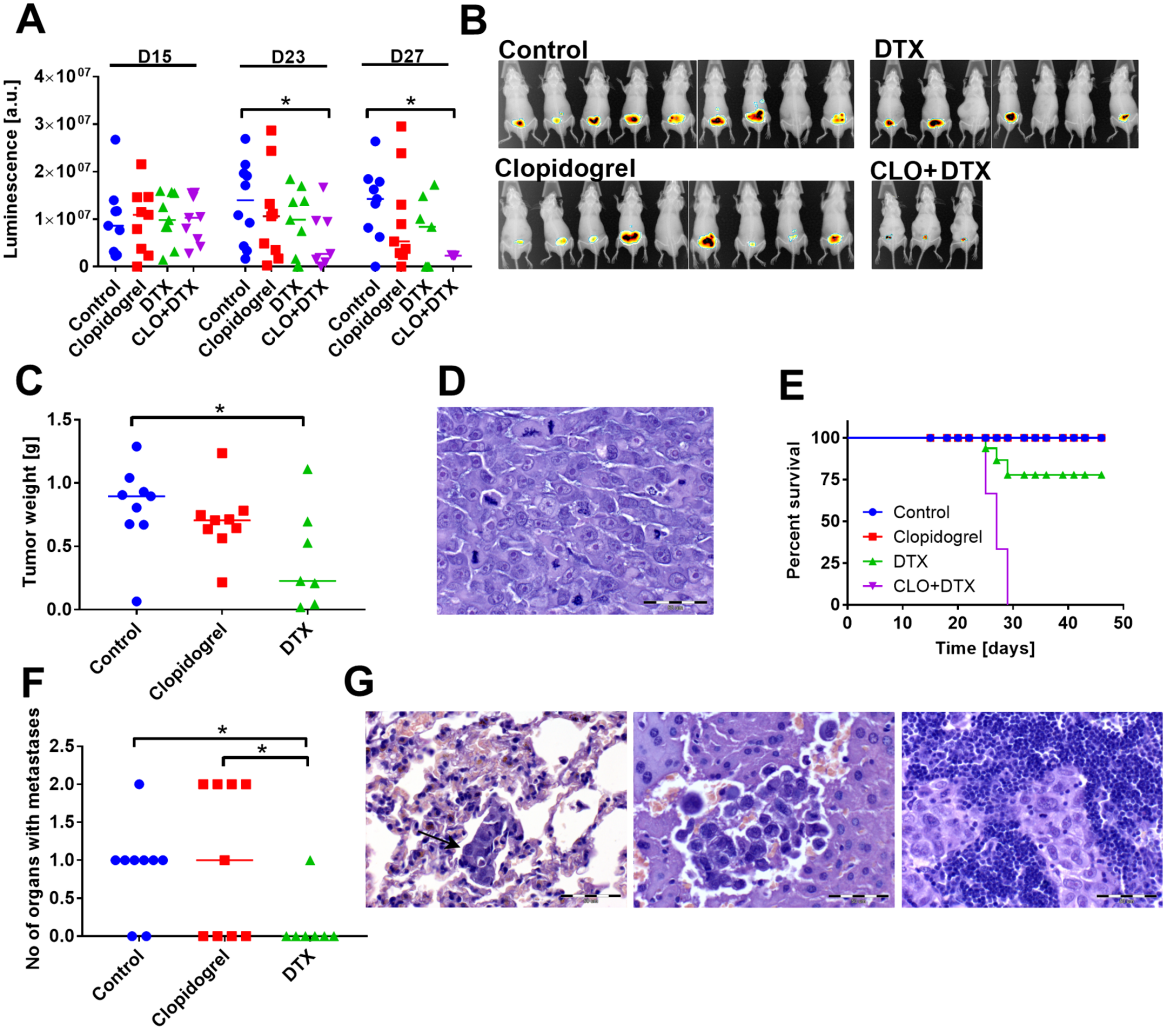
Clopidogrel increases the toxicity of docetaxel (DTX) in mice bearing human PC-3M-luc2 prostate tumors. (A) Luminescence of prostatic tumors on day 15 (day of treatment initiation), 23, and 27. (B) Photographs of mice on day 27 of experiment. (C) Tumor weight of tumors harvested on day 46. (D) Primary tumor tissue stained with hematoxylin and eosin (H&E) showing visible mitotic spindles. (E) Survival curves. (F) Number of organs with diagnosed metastases. (G) H&E-stained lung (left), liver (middle), and lymph node (right) tissue with micrometastases. All graphs show values for individual animals with median line with the exception of (E) shows percent of survival in indicated days. All mice were euthanized on day 46; the number of animals euthanized before: 12. N = 9 mice per group. Statistical analysis: Kruskal–Wallis test for multiple comparisons, *p<0.05. Survival analysis: Mantel–Cox test showed significant differences between survival curves (*p*<0.0001).

Combined treatment with clopidogrel and DTX significantly decreased the growth of primary tumors (Fig 7A). Because of an increased toxicity of such combined therapy (as presented in survival curves, Fig 7E), the antimetastatic activity of the proposed therapeutic regimen could not been assessed. Clopidogrel did not affect the metastatic dissemination of PC-3M-luc2 cells, but in mice treated with DTX, metastases were detected in less number of organs (Fig 7F and 7G).

**Fig 8:**
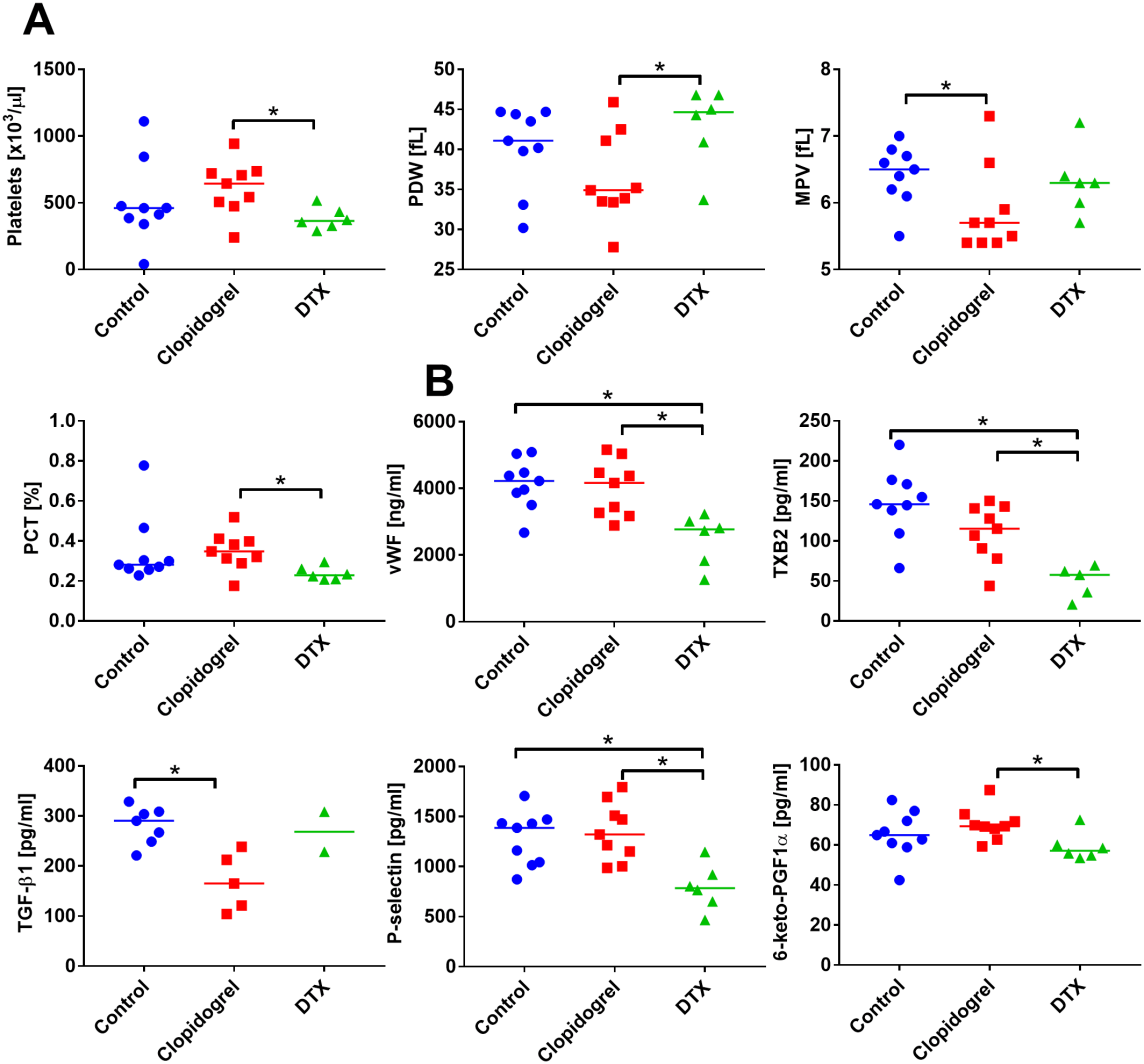
Platelet morphological parameters in PC-3M-luc2 bearing mice treated with clopidogrel combined with DTX. (A) Platelet morphological parameters, including platelet count, platelet distribution width (PDW), mean platelet volume (MPV), and plateletcrit (PCT). (B) ELISA measurements of plasma proteins related to platelets activity: von Willebrant factor (vWF), thromboxane b2 (TXB2), transforming growth factor beta 1 (TGF-β1), P-selectin, and prostacyclin metabolite (6-keto-PGF1α). All graphs show values for individual animals with median line. Plasma from 2 to 9 mice per group was analyzed. Statistical analysis: Kruskal–Wallis test for multiple comparisons; *p<0.05. The values of selected platelet parameters for healthy nude mice: platelet count: 525 ± 30 [×10^3^/μL]; MPV: 5.0 ± 0.3 [fL]; PDW: 31 ± 5 [fL]; PCT: 0.3 ± 0.03 [%].

Platelet count and PCT decreased in response to the treatment with DTX (Fig 8A). MPV was found to be diminished significantly by clopidogrel and was not affected by DTX, whereas PDW was found to be decreased by clopidogrel and increased by DTX (Fig 8A). Plasma concentration of vWF, TXB2, and P-selectin were significantly decreased by DXT. Clopidogrel significantly decreased plasma level of TGF-β1. Prostacyclin metabolite 6-keto-PGF1α was found to be unchanged (Fig 8B).

Combined treatment did not affect the body weight of mice significantly as compared to the treatment with DOX, CDDP, or TMX alone (S4A Fig).

The body weight of animals decreased during experiment because of tumor progression (S9A Fig). Total leucocyte count decreased significantly as a result of treatment with clopidogrel (the exception was monocytes) or DXT (S9B and S9D Fig). Furthermore, erythrocyte count and hemoglobin decreased after treatment with both agents. DTX decreased hematocrit and clopidogrel decreased MCHC significantly (S9C and S9E Fig).

Among blood biochemical parameters (LDH, AST, ALT, creatinine, urea, and glucose), only glucose was found to be significantly increased by DTX. The changes in creatinine level were not statistically significant, but the tendency to lower this parameter was observed after treatment with both agents (S10A Fig).

A pronounced decrease in the weight of kidneys and lungs were noticed in mice treated with clopidogrel or DTX. Moreover, DTX decreased the weight of spleen significantly. The weight of liver was unaffected by the treatment (S10B Fig).

### Combination therapy with clopidogrel and 5-FU in the subcutaneous model of mouse colon cancer and murine model of experimental metastasis of human colon cancer

#### Clopidogrel reveals no effect on the efficacy of 5-FU in the subcutaneous model of mouse colon cancer

**Fig 9:**
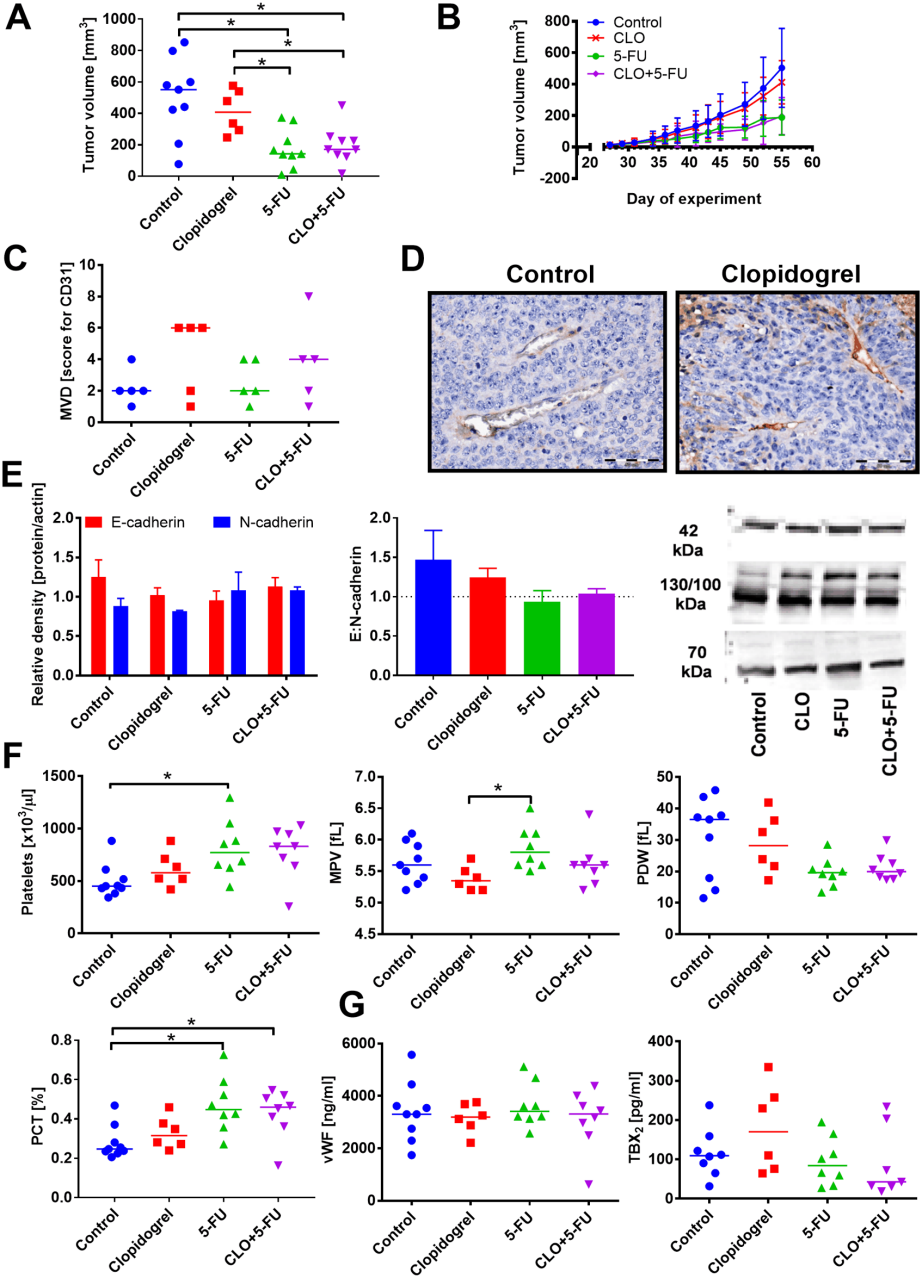
Clopidogrel did not affect tumor growth and metastasis in the combination therapy with 5-FU of mice bearing MC38/EGFP subcutaneous tumors. (A) Tumor weight on day 53. (B) Kinetics of tumor growth. (C) Score for microvessel density (MVD) measured in tumor tissue sections stained with anti-CD31 antibody. (D) Microphotographs of tumor tissue stained with anti-CD31 antibody from control and clopidogrel treated mice. (E) Expression of E- and N-cadherin in tumor tissue (left graph), E:N-cadgherin ratio (middle) and representative blots (right). (F) Morphological parameters of platelets, including platelet count, platelet distribution width (PDW), mean platelet volume (MPV), and plateletcrit (PCT). (G) ELISA measurements of plasma proteins connected with platelets activity: von Willebrant factor (vWF) and thromboxane B2 (TXB2). All graphs show values for individual animals with median line; the exception is panel E: mean with standard error of mean (SEM) and B: mean with standard deviation (SD) is presented. All mice were euthanized on day 53. N = 6–9 mice per group; some tests were performed on tissue or plasma from selected animals from each group (at least 3 for western blot; data for individual blots presented in S1 Table). Statistical analysis: Kruskal–Wallis test for multiple comparisons; *p<0.05. The values of selected platelet parameters for healthy C57Bl/6 mice: platelet count: 571 ± 94 [×10^3^/μL]; MPV: 5.4 ± 0.2 [fL]; PDW: 41 ± 4 [fL]; PCT: 0.3 ± 0.04 [%].

The volume of MC38/EFGP tumors decreased significantly in response to the treatment with 5-FU. The observed antitumor activity of chemotherapeutic agent was sustained in mice receiving the combined therapy with clopidogrel (Fig 9A and 9B). Accordingly, in the tumor tissue of the treated animals, we observed a decreased ratio of E:N-cadherin level (Fig 9E). Moreover, clopidogrel given alone as well as in combination with 5-FU tended to increase MVD in MC38/EGFP tumors (Fig 9C and 9D). Similar to the results obtained for BALB/c mice bearing 4T1 tumors, platelet count was increased in mice treated with 5-FU; however, clopidogrel did not affect their count. PDW, PCT, and MPV showed analogous changes such as in BALB/c mice (Fig 9F). However, the plasma level of vWF factor remained unchanged, whereas TXB2 level in MC38/EGFP bearing mice increased after clopidogrel treatment and decreased in combined treatment with 5-FU (*p*>0.05, Fig 9G).

Neither tumor growth nor the treatment applied affected the body weight of mice bearing MC38/EGFP tumors (S11A Fig). Similarly, MC38/EGFP tumors did not affect lymphocyte count, thus the effect of the proposed combined therapy on leukocyte count was less pronounced than in 4T1-tumor bearing mice. As shown in S11B and S11D Fig, 5-FU increased total leukocyte and granulocyte count (*p*<0.05). In general, morphological parameters of erythrocytes remained similar between all experimental groups, with the only exceptions for hematocrit that was increased by clopidogrel and MCHC decreased by 5-FU (S11C and S11E Fig).

The analysis of plasma biochemistry showed a significant change in urea concentration decreasing upon the treatment with 5-FU alone or in combination with clopidogrel (S12A Fig). In addition, in mice bearing MC38/EGFP tumors, the treatment with clopidogrel or 5-FU alone or in combination did not affect the weight of liver or spleen (S12B Fig).

#### Clopidogrel enhanced antitumor activity of 5-FU in the model of experimental metastasis of human colon cancer

**Fig 10:**
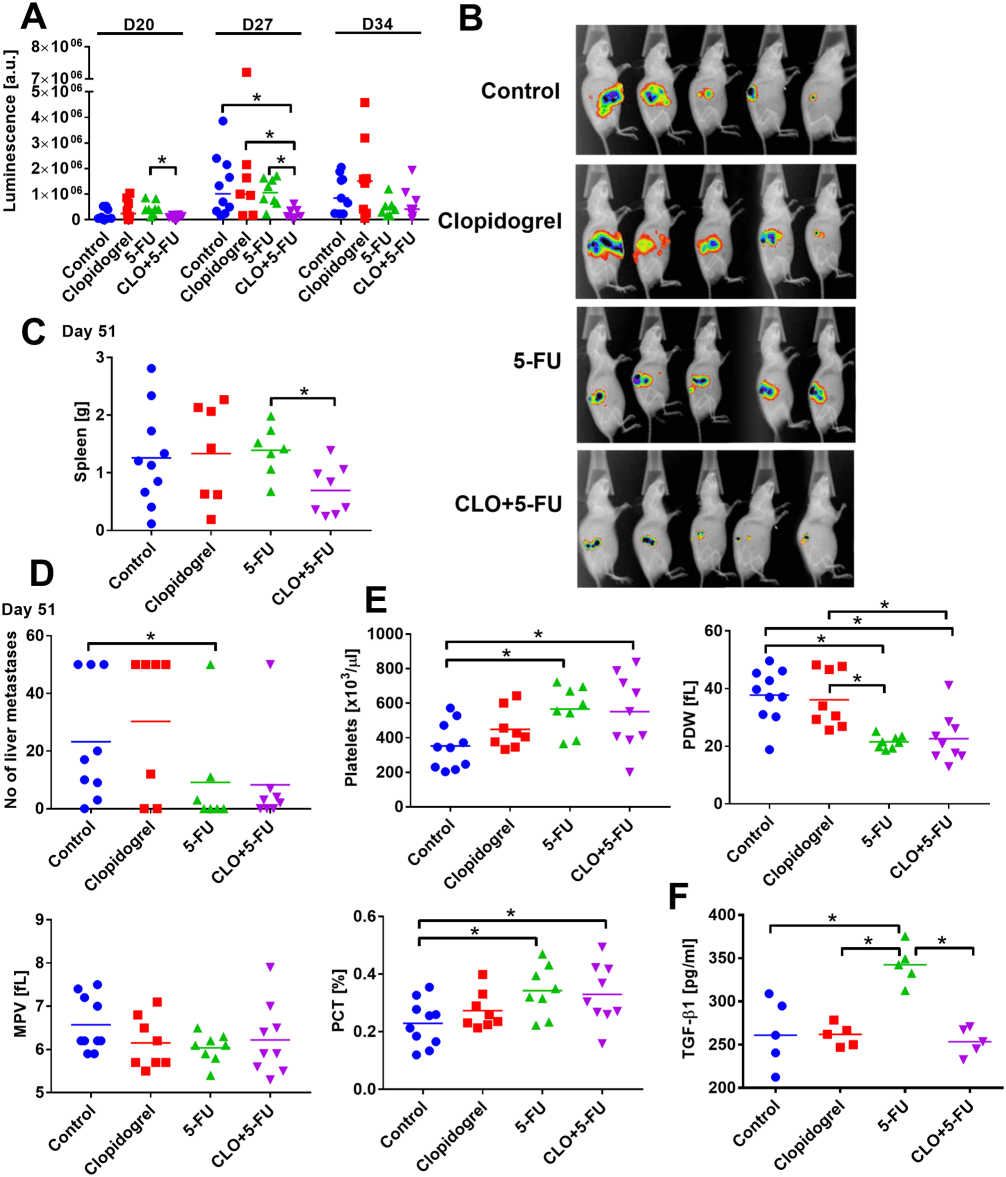
Clopidogrel improved tumor growth inhibition of 5-FU in mice transplanted intrasplenically with HT-29-luc2 human colon cancer. (A) Luminescence of intrasplenic tumors measured on days 20, 27, and 34. (B) Photographs of luminescence on day 27 of experiment. (C) Weight of spleens with primary tumors harvested on day 51. (D) Number of metastatic foci counted in the liver harvested on day 51. (E) Morphological parameters of platelets, including platelet count, platelet distribution width (PDW), mean platelet volume (MPV), and plateletcrit (PCT). (F) Plasma level of transforming growth factor β1 (TGF-β1). All graphs show values for individual animals with median line. All mice were euthanized on day 51. N = 7–10 mice per group (A–E); TGF-β1 was measured in plasma from five randomly selected mice (F). Statistical analysis: Kruskal–Wallis test for multiple comparisons; *p<0.05.

As evidenced by the measurements of luminescent signal originating from the intrasplenically transplanted HT-29-luc2 cells, combination therapy with clopidogrel and 5-FU resulted initially in the decrease in the tumor volume in the treated animals. But, this effect was not as pronounced on the latter days of the study (Fig 10A–10C). The weight of tumors growing within the spleen tissue measured on day 51 after transplantation was significantly lower in mice treated with combined treatment. The number of liver metastases was significantly lower only in mice treated with 5-FU (*p*<0.05 vs. control, Fig 10D). Furthermore, 5-FU alone or in combination with clopidogrel, significantly increased platelet count and consequently increased PCT, whereas it decreased PDV (Fig 10E). Moreover, 5-FU alone significantly increased the plasma level of TGF-β1, but when combined with clopidogrel, TGF-β1 levels were restored to the control values (Fig 10F).

We did not observe any adverse effects of the treatment as assessed based on the body weight changes as well as leukocyte morphology (S13A, S13B and S13D Fig). In mice treated with the combination therapy, fewer erythrocytes were observed (S13C Fig). Other RBC parameters such as hemoglobin level, MCHC as well as MCV, MCH, and RDW were decreased as result of treatment with 5-FU alone or when combined with clopidogrel (*p*<0.05) (S13E Fig). In addition, in mice treated with combination therapy, LDH, ALT, and bilirubin were significantly decreased (S14B Fig).

## Discussion

Clopidogrel, a thienopyridine derivative with antiplatelet activity, is widely used in patients with cardiovascular disease. The randomized controlled trial (TRITON-TIMI 38) comparing the efficacy of a novel thienopyridine, prasugrel with clinical outcome of clopidogrel in patients with acute coronary syndrome showed accelerated cancer progression (breast, colorectal, and prostate) in prasugrel and, to a lesser extent, clopidogrel arm [26,27]. However, further large population-based studies on cohorts of patients with colorectal, breast, and prostate cancer did not provide any evidence of an increased risk of cancer-specific mortality among patients using clopidogrel [28]. Other systematic review and meta-analysis studies have shown that thienopyridine exposure is not significantly associated with malignancy [29]. But, some studies suggest that clopidogrel even reduces cancer incidence [30]. Moreover, Sitia et al. in mouse model of chronic hepatitis reported the preventive activity of clopidogrel against hepatocellular carcinoma [31]. These findings were further supported by clinical trial data indicating that patients with hepatitis B virus-related hepatocellular carcinoma that were treated with clopidogrel showed improved recurrence-free survival and overall survival after liver resection [32].

In this study, we focused on three tumor models: breast, colon, and prostate cancer in which the use of thienopyridines was shown to be related with an increased cancer progression [26,27]. Mezouar et al. clearly showed that clopidogrel used alone can significantly inhibit primary tumor growth and metastasis of pancreatic cancer [21]. In the tumor models used in this study, clopidogrel used alone affected neither primary tumor growth nor metastasis or only tended to inhibit both parameters (Table 2). However, interesting observations were shown when clopidogrel was used in the combination therapy with various anticancer agents. As summarized in Table 2, in the combined treatment of 4T1 mouse mammary gland cancer with CP or with 5-FU, an increase in the anticancer and antimetastatic activity of both drugs was observed. Although clopidogrel alone tended to reduce the growth of MC38/EGFP mouse subcutaneous tumors, when the drug was administered simultaneously with 5-FU no improvement of cytostatic activity was observed. But, in human colon cancer model (HT-29-luc2) the growth of tumors transplanted intrasplenically was significantly diminished by the addition of clopidogrel to the treatment with 5-FU.

**Table 2:** Summarized effects of the combination therapy with clopidogrel and anticancer drugs on tumor growth and metastasis, as well as platelets condition.

Tumor model	Drug	Activity of combined treatment as compare to anticancer drug used alone^a^								
		Primary tumor size	Metastases	Platelets morphology				Blood biochemical parameters		
				PLT	PDW	MPV	PCT	vWF	TXB2	TGFβ
**4T1**	***CLO***	-	-/↓	-/↑	-/↓	-	-	-	-	↓↓
	CP	↓↓	-/↓	↑	-	-	-	-/↑	↓↓	NT
	CDDP	-	↑↑	-	↓↓	-	-	NT	NT	↓↓
	TMX	-/↓	↑	↑	↓	↓	-/↑	NT	NT	-
	DOX	↑	↑↑	↑	↓	-/↑	↑↑	NT	NT	↑↑
	5-FU	↓	↓	↓↓	↓	↓	-	-/↓	-/↑	↑↑
**MC38/EGFP**	***CLO***	↓	**NT**	-/↑	-/↓	-/↓	-/↑	-	-/↑	**NT**
	5-FU	-	NT	-	-	-/↓	-	-	-/↑	NT
**HT-29-luc2**	***CLO***	-	-	-/↑	-	-/↓	-/↑	**NT**	**NT**	-
	5-FU	↓↓	-	-	-	-	-	NT	NT	↓↓
**PC-3M-luc2**	***CLO***	-/↓	-	-/↑	-/↓	↓↓	-	-	-/↓	↓↓
	DTX	↓	NT’	NT’	NT’	NT’	NT’	NT’	NT’	NT’
	MTX*	-/↓	↓	-	-	-/↑	-	NT’	NT’	-

^a^In case of CLO—as compared to control mice;

NT—not tested, NT’—not tested because of animals death; ↑—an increase (e.g., an increase in number of metastases, tumor size, or platelet count); ↓—a decrease; -/↓ or -/↑—tendency observed; ↑↑ or ↓↓—statistically significant changes;—no effect.

*data from experiment with lowered doses of MTX.

**Abbreviations**: PLT, platelet count; PDW, platelet distribution width; MPV, mean platelet volume; PCT, plateletcrit; vWF, von Willebrand factor; TXB2, thromboxane B2; TGFβ, transforming growth factor β; CLO, clopidogrel; MTX, mitoxantrone.

The observed enhanced antitumor activity of the combined therapy, most pronounced in case of the treatment comprising CP, could be related to the increase in E:N-cadherin ratio observed in tumor tissue. Such enhanced expression of E-cadherin versus N-cadherin may be one of the signs of diminished EMT. EMT is a process known to endow the tumor cells with invasive mesenchymal-like phenotype and to be driven by platelet-derived TGF-β, among others. Acceleration of EMT and direct platelet-tumor cell contact synergistically activate the TGF-β/Smad and NF-κB pathways in cancer cells, resulting in their enhanced invasion and consequently *in vivo* metastasis [12]. The mechanism of antimetastatic activity observed in the combined treatment with 5-FU in 4T1 cancer model can be explained by the decrease in invasive potential of tumor cells (reflected in the increased E:N-cadherin ratio) and by the decrease in the accumulation of platelets in the tumor [21]. In addition, the decrease in platelet activation, through diminished vWF plasma concentration [33] in mice treated with both 5-FU and clopidogrel can contribute to antimetastatic effect of the combined treatment.

However, in the discussed 4T1 mammary gland cancer model, we also observed some undesirable effects of clopidogrel used in combination treatment with CDDP, TMX, and DOX leading to a significant increase in the formation of lung metastatic foci in the treated mice. This is contrary with the work of Pandey et al. who reported an enhanced antitumor activity of CDDP or CDDP nanoparticles administered with clopidogrel. Other authors also showed that 4T1 cells expressed P2Y12 receptors and when co-incubated *in vitro* with CDDP and P2Y12 inhibitor, significantly higher cytotoxic response in 4T1 cancer cell line was observed. However, CDDP in these studies increased P2Y12 receptor expression on cancer cells [34], which was found to be disadvantageous, because P2Y12 receptors agonists were shown to stimulate the proliferation of glioma cells [35,36].

According to Pandey et al., higher efficacy of chemotherapy with CDDP was caused by the increased vessel permeability induced by clopidogrel and the resulting improved drug delivery [10]. But, according to our results, the blood vessel permeability in 4T1 tumors in mice treated with clopidogrel was similar to that observed in control tumors. Furthermore, similar permeability was observed for tumors treated with CP and CP combined with clopidogrel. Moreover, MVD estimated by immunohistological staining of CD31 molecule showed that upon treatment with clopidogrel, MVD was found to be increased in MC38/EGFP or contrarily slightly decreased in PC-3M-luc2 tumors. The intratumoral vessels’ network might also be built up by tumor cells [37,38]. Such vasculogenic mimicry was shown in small cell lung cancer xenografts to decrease tumor latency, and despite an increased intra-tumor delivery of CDDP, it decreased CDDP efficacy [38]. Moreover, in mice bearing orthotopic 4T1 tumors vascularized by vessels built up by tumor cells that were treated with anticoagulant warfarin, an increased metastatic potential was observed [37]. Therefore, increased leakage of tumor vasculature can lead not only to a better drug penetration but also to the decreased drug efficacy and increased metastatic potential of cancer cells.

However, Yang et al. have shown that clopidogrel protects endothelium against oxidative stress and consequently diminish endothelium dysfunction [39]. Therefore, depending on tumor model, clopidogrel can affect tumor vasculature by influencing platelet activity [7,8,40] or can directly interfere with endothelium by modulating its activation status [39]. These effects, depending on the changes in tumor microenvironment induced by anticancer drug, may be one of the mechanisms of the observed improvement or deterioration of the antitumor activity of antineoplastic agents given in the combination therapy with clopidogrel.

Platelet granules contain a variety of factors, such as VEGF, TGF-β, and so on that are secreted upon platelet activation. Many of these factors have been implicated in various steps of tumor progression and metastasis. Indeed, due to the tumor vasculature leakage, platelets may come in direct contact with tumor cells and deposit several of angiogenic factors *in situ*, thus promoting tumor vascularization [7]. But, our data show that in mice treated with clopidogrel, the level of serum proteins related to platelet activity changed between cancer models and anticancer drugs applied in the combined treatment. Only TGF-β1 plasma concentration was diminished by clopidogrel alone in mice bearing mammary gland as well as prostate cancer. Elevated TGF-β plasma level is proposed as a marker of metastatic breast cancer with poor prognosis [41]. Moreover, TGF-β is known to modulate regulatory T-cell (Treg) differentiation, which suppress antitumor T cell response [42]. TGF-β also promotes the differentiation of tumor-promoting tumor-associated macrophages (M2), and tumor-associated neutrophils (N2) [43,44]. Significant increase in TGF-β1 plasma level was observed in our study in mice bearing 4T1 mammary gland tumors treated with clopidogrel combined with DOX or with 5-FU. It correlates with the significant increase in metastases number observed in mice treated with clopidogrel combined with DOX and can explain such unfavorable effect. But, it has been reported that in women with an early stage, pre-metastatic cancer, elevated levels of TGF-β1 are observed [45]. This is in agreement with our observations on 4T1 tumor-bearing mice that were treated with clopidogrel and 5-FU, which indicates that in these animals, tumor progression was markedly but probably reversibly inhibited. In human colon cancer model, clopidogrel decreased the level of TGF-β1 that was elevated by 5-FU which in turn contributed to the antitumor effect of combined treatment.

While analyzing the adverse effects resulting from the use of the combination therapies in mice bearing 4T1 tumors, we only observed the decrease in body weight in the animals treated with the combined therapy with TMX. However, LDH, alanine aminotransaminase (ALT), and aspartate aminotransaminase (AST) levels in these animals decreased suggesting that overall the use of clopidogrel in the combination therapies does not impair treatment safety. Significant increase in treatment toxicity was observed when clopidogrel was used simultaneously with DTX in nude mice bearing prostate tumors. The metabolism of taxanes, such as paclitaxel or DTX is related to the hepatic cytochrome (CYP) P450 3A isoform CYP3A4 [46]. DTX can be metabolized by the CYP3A5 [47], whereas paclitaxel can be metabolized by CYP2C8 [46]. Similarly, clopidogrel is considered to be primarily the substrate for CYP3A4/5 [48], whereas its metabolite acyl-β-D-glucuronide is a potent inhibitor of CYP2C8 [49]. A published case report as well as larger epidemiological studies indicate that clopidogrel is associated with a clinically relevant increased risk of neuropathy in patients treated with paclitaxel [49,50]. In the cited report, authors suggested that clinically used doses of clopidogrel can reduce the CYP2C8-mediated systemic clearance of paclitaxel, leading to an increased risk of paclitaxel toxicity [50]. Since TMX, DTX, and clopidogrel are all CYP3A4 substrates [47,48,51] and are characterized by a mild to substantial toxicity, their concurrent use with clopidogrel may lead to the development of severe toxicities, as observed in our studies. Such hypothesis may be further supported by the results of the studies showing that clopidogrel incubation with CYP3A4 led to the formation of metabolites that are toxic for hepatocytes and can be trapped by glutathione. However, clopidogrel depletes the cellular glutathione pool. Thus, high CYP3A4 activity and low cellular glutathione supplies may be the risk factors for clopidogrel-associated hepatocellular toxicity [52]. Low glutathione level may also increase the toxicity of DTX or other anticancer drugs being substrates of this detoxification system [53]. Likewise, metabolic pathway and toxicity of CP, even when used in low doses, are also related to the liver function [54]. Although we did not observe any clinical signs of an increased toxicity of the combined treatment with clopidogrel and CP, significant increase in LDH level as compared to CP alone was observed in serum of mice treated with both agents, suggesting increased hepatic toxicity of such treatment.

Leukopenia, in addition to toxic neurological effects, is the primary toxic effect of DTX [47]. But, in human neutrophils, granulocytes, and lymphocytes, clopidogrel has been shown to decrease the membrane potential of the inner mitochondrial membrane, increase ROS production, and induce cytochrome-c release into the cytoplasm ultimately leading to apoptosis [55]. Therefore, leukocytes affected by anticancer drugs may be more susceptible to apoptotic process during combined treatment with clopidogrel. Accordingly, in mice bearing PC-3M-luc2 prostate tumors, clopidogrel significantly decreased leukocyte count, which was caused by the reduction of lymphocyte and granulocyte count (summarized in Table 3). Similar tendency after clopidogrel treatment was observed in the same strain of mice (nude mice) bearing HT29-luc2 tumors. Therefore, we can speculate that in mice treated with both clopidogrel and DTX, this effect was further accelerated, contributing to the observed toxic effects of the combined treatment. However, in mice bearing HT-29-luc2 or MC38/EGFP colon tumors that were treated with 5-FU, blood parameters did not change significantly; therefore, the effect of clopidogrel on blood cell count in the combined treatment with 5-FU was also not significant.

**Table 3:** Summary of toxicological parameters in mice treated with the studied combination therapies.

Tumor model	Drug	Body weight/survival	Blood morphology					Blood biochemical parameters				
			RBC	Leuk.	Lym.	Mon.	Gra.	LDH	AST	ALT	CRE	URE
**4T1**	***CLO***	-	- ***or*** ↓↓^a^	-/↑	↑↑	↑↑	↑	-	-/↑	-/↑	-/↓	↓
	CP	-	-	-/↑	-/↓	-	-/↑	↑↑	-/↑	-	-/↓	NT
	CDDP	-	-	-/↓	↓	↓	-/↓	-	-	-	↓↓	-
	TMX	-/↓	↑↑	-	-	-	-	↓↓	↓	↓↓	↓↓	-
	DOX	-	↑↑	↑↑	↑↑	↑↑	↑↑	-	↑↑	-	↓	-
	5-FU	-	-	↑	↑↑	↑	-	-	-/↑	-	-/↓	-
**MC38/EGFP**	***CLO***	-	- ***or*** ↑↑^a^	-	-	-	-/↓	-	-	-	-	-/↓
	5-FU	-	-	↓	-/↓	-	↓	-/↑	-	-	-	-
**HT-29-luc2**	***CLO***	-	-	-/↓	-/↓	-/↓	-/↓	-	-	-	-	-
	5-FU	-	-	-	-/↑	-	-/↓	↓↓	-	↓↓	-	-/↓
**PC-3M-luc2**	***CLO***	-	↓↓	↓↓	↓↓	-/↓	↓↓	-	-	-/↓	-/↓	-
	DTX	↓↓	NT’	NT’	NT’	NT’	NT’	NT’	NT’	NT’	NT’	NT’
	MTX*	↑↑	-	-	-/↑	-/↑	-/↑	-/↓	-	-	↑	-/↓

The activity of the combined treatment is shown as compared to anticancer drug used alone or in the case of CLO—as compared to control mice.

↓↓^a^ in case of MCH and MCHC;

↑↑^a^ in case of hematocrit;

NT—not tested, NT’—not tested because of animals death; ↑—an increase (e.g. an increase in number of metastases, tumor size, or platelet count); ↓—a decrease; -/↓ or -/↑—tendency observed; ↑↑ or ↓↓—statistically significant changes;—no effect.

* data from experiment with lowered doses of MTX.

**Abbreviations**: RBC, red blood cell; Leuk, leukocyte; Lym, lymphocyte; Mon, monocyte; Gra, granulocyte; LDH, lactate dehydrogenase; AST, aspartate aminotransaminase; ALT, alanine aminotransaminase; Cre, creatinine; Ure, urea; CLO, clopidogrel; MCH, mean corpuscular hemoglobin; MCHC, mean corpuscular hemoglobin concentration; MTX, mitoxantrone.

Different effects of clopidogrel on leukocyte were observed in 4T1 tumor model, a highly metastatic and highly immunogenic mammary gland cancer. Its rapid progress is followed by leukocytosis caused by the expansion of all leukocyte populations and accompanying splenomegalia [56]. In this tumor model, clopidogrel alone increased the leukocyte count significantly, but its effects in the combined treatment varied according to the anticancer drug used. In the combined treatment of clopidogrel with CP and with CDDP, a decrease in lymphocyte and monocyte count was observed, whereas with DOX, 5-FU, and to a lesser extent with TMX, significant increase in leukocyte was observed as compared to the treatment with anticancer drug alone and/or to control 4T1 tumor-bearing mice. Although leukocytes can contribute to cancer growth and metastasis [57], there was no correlation between the effect on leukocyte and the pro- or antimetastatic effect of the combined treatment applied. In various diseases, the direct interaction of platelets and leukocytes lead to the targeted release of soluble mediators and reciprocal activation of both cell types. Depending on the environment, platelet–leukocyte interplay can boost and dampen inflammatory responses [58]. For example, different effects of aspirin on platelet-inflammatory biomarkers are observed in healthy volunteers when compared to patients with acute ischemic stroke [59].

In this study with prostate cancer model, clopidogrel was found to protect against the MTX-induced toxicity. MTX and DOX affect the heart by impairing mitochondrial function. Both drugs share similarities in their modes of toxicity as redox-interfering drugs and inducers of energy imbalance [60]. The decreased toxicity of MTX due to clopidogrel may be related to the observed antioxidant properties of clopidogrel in patients with coronary heart disease [61]. Moreover, clopidogrel improves endothelial nitric oxide bioavailability [62] as well as activate defensive mechanisms of endothelium involving NO- and prostacyclin PGI_2_-mediated actions [63]. These activities may protect vascular wall against toxic effects of MTX.

### Conclusion

Enhanced platelet activity that accompanies the development of malignant tumor is not only a risk factor for cardiovascular events but also contributes to metastases formation. Therefore, the use of antiplatelet agents in the combined treatment of metastatic cancer may become an important strategy in controlling both the venous thromboembolism and metastasis in patients with cancer. As we have shown in this study, clopidogrel can increase anticancer and antimetastatic activity of some of the known anticancer drugs, such as 5-FU, CP, or MTX, whereas it can decrease the efficacy of the other anticancer drugs, such as DOX, CDDP, and TMX. Moreover, clopidogrel increases the toxicity of DTX, whereas it protects against toxicity of MTX. Observed effects may be related to various aspects of clopidogrel activities including its primary target—platelets and others such as endothelial cells, leukocytes, and cancer cells; however, further studies are needed to exactly explain the role of each one. These observations suggest that antiplatelet agents are an important player in the anticancer treatment, but their use in patients should be carefully reviewed to avoid undesirable drug interactions.

## Supporting information

S1 Fig General toxicity parameters in mice bearing 4T1 tumors treated with clopidogrel combined with 5-fluorouracil (5-FU) measured as body weight kinetics and blood morphology. (A) Body weight kinetics. (B) Leukocyte count. (C) Erythrocyte count. (D) Leukocyte population: lymphocyte, monocyte, and granulocyte count. (E) Blood morphological parameters characteristic for erythrocytes: hematocrit, hemoglobin, mean corpuscular volume (MCV), mean corpuscular hemoglobin (MCH), mean corpuscular hemoglobin concentration (MCHC), and red (cell) distribution width (RDW). All graphs show values for individual animals with median line; the exception: (A) the mean body weight ± standard deviation (SD) is presented. N = 10 mice per group; some tests were performed on blood from selected animals from each group. Statistical analysis: Kruskal–Wallis test for multiple comparisons; *p<0.05.
(TIF)

S2 Fig General toxicity parameters in mice bearing 4T1 tumors treated with clopidogrel combined with cyclophosphamide (CP) measured as body weight kinetics and blood morphology. (A) Body weight kinetics. (B) Leukocyte count. (C) Erythrocyte count. (D) Leukocyte population: lymphocyte, monocyte, and granulocyte count. (E) Blood morphological parameters characteristic for erythrocytes: hematocrit, hemoglobin, mean corpuscular volume (MCV), mean corpuscular hemoglobin (MCH), mean corpuscular hemoglobin concentration (MCHC), and red (cell) distribution width (RDW). All graphs show values for individual animals with median line; the exception: (A) the mean body weight ± standard deviation (SD) is presented. N = 10 mice per group; some tests were performed on blood from selected animals from each group. Statistical analysis: Kruskal–Wallis test for multiple comparisons; *p<0.05.
(TIF)

S3 Fig Blood biochemistry and selected organ weight in mice bearing 4T1 tumors treated with clopidogrel combined with 5-fluorouracil (5-FU) or cyclophosphamide (CP). (A) Blood biochemical parameters in mice treated with clopidogrel combined with 5-FU: aspartate aminotransferase (AST), alanine transaminase (ALT), albumin, creatinine, and urea. (B) The weight of spleen, kidneys, and liver of mice treated with clopidogrel and 5-FU. (C) The weight of liver, spleen, and kidneys of mice treated with clopidogrel and CP. All graphs show values for individual animals with median line. N = 10 mice per group; some tests were performed on plasma from selected animals from each group. Statistical analysis: Kruskal–Wallis test for multiple comparisons; *p<0.05.
(TIF)

S4 Fig General toxicity parameters in mice bearing 4T1 tumors treated with clopidogrel combined with doxorubicin (DOX), cisplatin (CDDP), and tamoxifen (TMX) measured as body weight kinetics and leukocytes morphology. (A) Body weight kinetics, (B) leukocyte count, (C) lymphocyte count, (D) monocyte count, and (E) granulocyte count. All graphs show values for individual animals with median line; the exception: (A) the mean body weight ± standard deviation (SD) is presented. N = 10–12 mice per group; some tests were performed on blood from selected animals from each group. Statistical analysis: Kruskal–Wallis test for multiple comparisons; *p<0.05.
(TIF)

S5 Fig Erythrocyte morphology in mice bearing 4T1 tumors treated with clopidogrel combined with doxorubicin (DOX), cisplatin (CDDP), and tamoxifen (TMX). (A) Erythrocyte count. (B) Hemoglobin. (C) Hematocrit. (D) Mean corpuscular volume (MCV). (E) Mean corpuscular hemoglobin (MCH). (F) Mean corpuscular hemoglobin concentration (MCHC). (G) Red (cell) distribution width (RDW). All graphs show values for individual animals with median line. N = 10–12 mice per group; some tests were performed on blood from selected animals from each group. Statistical analysis: Kruskal–Wallis test for multiple comparisons; *p<0.05.
(TIF)

S6 Fig Weight of selected organs and blood biochemical parameters in mice bearing 4T1 tumors treated with clopidogrel combined with doxorubicin (DOX), cisplatin (CDDP), and tamoxifen (TMX). The weight of (A) liver, (B) spleen, and (C) kidneys. The level of (D) alanine aminotransferase (ALT), (E) aspartate aminotransaminase (AST), (F) creatinine, and (G) urea. N = 8–10 mice per group; some tests were performed on blood from selected animals from each group. All graphs show values for individual animals with median line. Statistical analysis: Kruskal–Wallis test for multiple comparisons; *p<0.05.
(TIF)

S7 Fig General toxicity parameters in mice bearing PC-3M-luc2 tumors treated with clopidogrel combined with mitoxantrone (MTX) measured as body weight kinetics and blood morphology. (A) Body weight kinetics. (B) Leukocyte count. (C) Erythrocyte count. (D) Leukocyte population: lymphocyte, monocyte, and granulocyte count. (E) Blood morphological parameters characteristic for erythrocytes: hematocrit, hemoglobin, mean corpuscular volume (MCV), mean corpuscular hemoglobin (MCH), mean corpuscular hemoglobin concentration (MCHC), and red (cell) distribution width (RDW). (F) The weight of liver, spleen, and kidneys. (G) Microvessel density (MVD) scored as CD31 immunohistochemical staining. All graphs show values for individual animals with median line; the exception: (A) the mean body weight ± standard deviation (SD) is presented. A–F—MTX at the dose of 3 mg/kg/dose; G—1 mg/kg/dose. N = 7–9 mice per group; some tests were performed on tissue from selected animals from each group. Statistical analysis: Kruskal–Wallis test for multiple comparisons; *p<0.05.
(TIF)

S8 Fig Blood biochemical parameters in mice bearing PC-3M-luc2 tumors treated with clopidogrel combined with mitoxantrone (MTX, 1 mg/kg/dose). The level of (A) lactate dehydrogenase (LDH), (B) creatine kinase (CK), (C) alanine aminotransferase (ALT), (D) aspartate aminotransferase (AST), (E) creatinine, and (F) urea. N = 5 mice per group. All graphs show values for individual animals with median line. Statistical analysis: Kruskal–Wallis test for multiple comparisons; *p<0.05.
(TIF)

S9 Fig General toxicity parameters in mice bearing PC-3M-luc2 tumors treated with clopidogrel combined with docetaxel (DTX) measured as body weight kinetics and blood morphology. (A) Body weight kinetics. (B) Leukocyte count. (C) Erythrocyte count. (D) Leukocyte populations: lymphocyte, monocyte, and granulocyte count. (E) Blood morphological parameters characteristic for erythrocytes: hematocrit, hemoglobin, mean corpuscular volume (MCV), mean corpuscular hemoglobin (MCH), mean corpuscular hemoglobin concentration (MCHC), and red (cell) distribution width (RDW). All graphs show values for individual animals with median line; the exception: (A) the mean body weight ± standard deviation (SD) is presented. N = 9 mice per group. Statistical analysis: Kruskal–Wallis test for multiple comparisons; *p<0.05.
(TIF)

S10 Fig Blood biochemistry and selected organ weight in mice bearing PC-3M-luc2 tumors treated with clopidogrel combined with docetaxel (DTX). (A) Blood biochemical parameters: lactate dehydrogenase (LDH), aspartate aminotransferase (AST), alanine transaminase (ALT), creatinine, urea, and glucose. (B) The weight of liver, spleen, and kidneys. All graphs show values for individual animals with median line. N = 9 mice per group. Statistical analysis: Kruskal–Wallis test for multiple comparisons; *p<0.05.
(TIF)

S11 Fig General toxicity parameters in mice bearing MC38/EGFP tumors treated with clopidogrel combined with 5-fluorouracil (5-FU) measured as body weight kinetics and blood morphology. (A) Body weight kinetics. (B) Leukocyte count. (C) Erythrocyte count. (D) Leukocyte populations: lymphocyte, monocyte, and granulocyte count. (E) Blood morphological parameters characteristic for erythrocytes: hematocrit, hemoglobin, mean corpuscular volume (MCV), mean corpuscular hemoglobin (MCH), mean corpuscular hemoglobin concentration (MCHC), and red (cell) distribution width (RDW). All graphs show values for individual animals with median line; the exception: (A) the mean body weight ± standard deviation (SD) is presented. N = 6–9 mice per group; some tests were performed on tissue or plasma from selected animals from each group. Statistical analysis: Kruskal–Wallis test for multiple comparisons; *p<0.05.
(TIF)

S12 Fig Blood biochemistry and selected organ weight in mice bearing MC38/EGFP tumors treated with clopidogrel combined with 5-fluorouracil (5-FU). (A) Blood biochemical parameters: lactate dehydrogenase (LDH), aspartate aminotransferase (AST), alanine transaminase (ALT), creatinine, urea, and glucose. (B) The weight of liver and spleen. All graphs show values for individual animals with median line. N = 6–9 mice per group; some tests were performed on tissue or plasma from selected animals from each group. Statistical analysis: Kruskal–Wallis test for multiple comparisons; *p<0.05.
(TIF)

S13 Fig General toxicity parameters in mice bearing HT29-luc2 tumors treated with clopidogrel combined with 5-fluorouracil (5-FU) measured as body weight kinetics and blood morphology. (A) Body weight kinetics. (B) Leukocyte count. (C) Erythrocyte count. (D) Leukocyte populations: lymphocyte, monocyte, and granulocyte count. (E) Blood morphological parameters characteristic for erythrocytes: hematocrit, hemoglobin, mean corpuscular volume (MCV), mean corpuscular hemoglobin (MCH), mean corpuscular hemoglobin concentration (MCHC), and red (cell) distribution width (RDW). All graphs show values for individual animals with median line; the exception: (A) the mean body weight ± standard deviation (SD) is presented. N = 8–9 mice per group. Statistical analysis: Kruskal–Wallis test for multiple comparisons; *p<0.05.
(TIF)

S14 Fig Blood biochemistry and selected organ weight in mice bearing HT29-luc2 tumors treated with clopidogrel combined with 5-fluorouracil (5-FU). (A) Blood biochemical parameters: lactate dehydrogenase (LDH), aspartate aminotransferase (AST), alanine transaminase (ALT), creatinine, urea, albumin, and bilirubin. (B) The weight of liver. All graphs show values for individual animals with median line. N = 8–9 mice per group. Statistical analysis: Kruskal–Wallis test for multiple comparisons; *p<0.05.
(TIF)

S1 Table Densitometric analysis of individual blots. (DOCX)

S1 File Tumor volume and body weights for individual mice during experiment course. The data for individual animals on body weight and tumor growth kinetics which are not included in the figures are collected in the separate sheets containing data for each type of cancer. CLO, clopidogrel; 5-FU, 5-fluorouracil; CP, cyclophosphamide; DOX, doxorubicine; TMX, tamoxifen; CDDP, cisplatin; MTX, mitoxantrone; DTX, docetaxel.
(XLSX)
