# Supplementary figures and images for: Clopidogrel in a combined therapy with anticancer drugs—effect on tumor growth, metastasis, and treatment toxicity: Studies in animal models
Source: PLoS One. 2017 Dec 5;12(12):e0188740. doi: 10.1371/journal.pone.0188740 (PMC5716579; doi:10.1371/journal.pone.0188740)

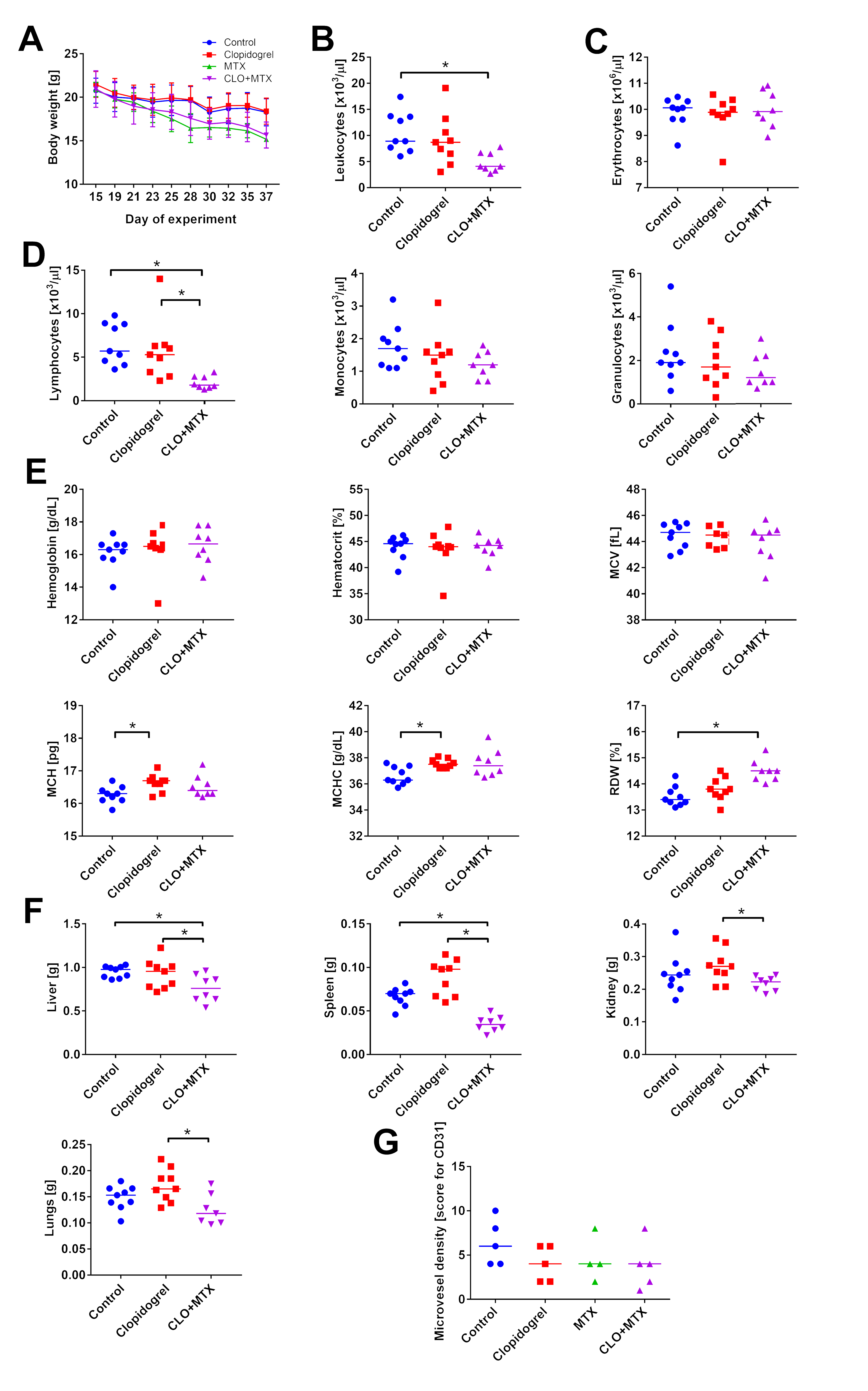

Supplement: S7 Fig — (A) Body weight kinetics. (B) Leukocyte count. (C) Erythrocyte count. (D) Leukocyte population: lymphocyte, monocyte, and granulocyte count. (E) Blood morphological parameters characteristic for erythrocytes: hematocrit, hemoglobin, mean corpuscular volume (MCV), mean corpuscular hemoglobin (MCH), mean corpuscular hemoglobin concentration (MCHC), and red (cell) distribution width (RDW). (F) The weight of liver, spleen, and kidneys. (G) Microvessel density (MVD) scored as CD31 immunohistochemical staining. All graphs show values for individual animals with median line; the exception: (A) the mean body weight ± standard deviation (SD) is presented. A–F—MTX at the dose of 3 mg/kg/dose; G—1 mg/kg/dose. N = 7–9 mice per group; some tests were performed on tissue from selected animals from each group. Statistical analysis: Kruskal–Wallis test for multiple comparisons; *p<0.05. (TIF) [file pone.0188740.s007.tif]
