# Supplementary material for: Clopidogrel in a combined therapy with anticancer drugs—effect on tumor growth, metastasis, and treatment toxicity: Studies in animal models
Source: PLoS One. 2017 Dec 5;12(12):e0188740. doi: 10.1371/journal.pone.0188740 (PMC5716579; doi:10.1371/journal.pone.0188740)
Supplement: S1 Table — (DOCX) [file pone.0188740.s015.docx]

S1 Table. Densitometric analysis of individual blots.

| Data location | Group | E-cadherin | | | N-cadherin | | | E:N cadherin ratio | | |
| --- | --- | --- | --- | --- | --- | --- | --- | --- | --- | --- |
|  |  | Blot 1 | Blot 2 | Blot 3 | Blot 1 | Blot 2 | Blot 3 | Blot 1 | Blot 2 | Blot 3 |
| Figure 1C | Control | 0.55 | 1.57 | 0.29 | 0.80 | 1.67 | 0.86 | 0.69 | 0.94 | 0.34 |
|  | Clopidogrel | 0.76 | 1.05 | 0.88 | 0.76 | 0.89 | 2.85 | 1.01 | 1.18 | 0.31 |
|  | 5-FU | 0.81 | 0.56 | 0.87 | 1.36 | 0.32 | 1.28 | 0.60 | 1.75 | 0.68 |
|  | CLO+5-FU | 0.63 | 0.48 | 1.16 | 1.12 | 0.54 | 0.26 | 0.56 | 0.89 | 4.38 |
| Figure 2E | Control | 1.10 | 0.69 | 6.39 | 0.79 | 1.35 | 9.92 | 1.38 | 0.51 | 0.64 |
|  | Clopidogrel | 3.49 | 0.66 | 9.67 | 3.61 | 0.83 | 3.30 | 0.97 | 0.79 | 2.93 |
|  | CP | 0.85 | 0.46 | 6.50 | 0.90 | 0.53 | 12.24 | 0.94 | 0.88 | 0.53 |
|  | CLO+CP | 1.24 | 1.70 | 12.21 | 0.90 | 0.65 | 1.64 | 1.38 | 2.63 | 7.46 |
| Figure 9E | Control | 0.88 | 1.64 | 1.24 | 0.84 | 0.74 | 1.07 | 1.04 | 2.21 | 1.16 |
|  | Clopidogrel | 1.07 | 1.15 | 0.83 | 0.80 | 0.83 | 0.82 | 1.34 | 1.38 | 1.02 |
|  | 5-FU | 1.00 | 1.14 | 0.73 | 1.52 | 1.01 | 0.72 | 0.66 | 1.13 | 1.02 |
|  | CLO+5-FU | 0.99 | 1.36 | 1.04 | 1.02 | 1.17 | 1.05 | 0.97 | 1.16 | 0.99 |

E-cadherin and N-cadherin contents were normalized to β-actin. Finally, E-cadherin to N-cadherin ratios in individual samples were calculated.
